# A transcription factor, PbWRKY24, contributes to russet skin formation in pear fruits by modulating lignin accumulation

**DOI:** 10.1093/hr/uhae300

**Published:** 2024-10-18

**Authors:** Jialong Wang, Dong Wang, Mingrui Zhao, Mengyuan Yu, Xiaodong Zheng, Yike Tian, Zhijuan Sun, Xiaoli Liu, Caihong Wang, Changqing Ma

**Affiliations:** College of Horticulture, Qingdao Agricultural University, Qingdao 266109, China; Engineering Laboratory of Genetic Improvement of Horticultural Crops of Shandong Province, Qingdao 266109, China; College of Horticulture, Qingdao Agricultural University, Qingdao 266109, China; Engineering Laboratory of Genetic Improvement of Horticultural Crops of Shandong Province, Qingdao 266109, China; College of Horticulture, Qingdao Agricultural University, Qingdao 266109, China; Engineering Laboratory of Genetic Improvement of Horticultural Crops of Shandong Province, Qingdao 266109, China; College of Horticulture, Qingdao Agricultural University, Qingdao 266109, China; Engineering Laboratory of Genetic Improvement of Horticultural Crops of Shandong Province, Qingdao 266109, China; College of Horticulture, Qingdao Agricultural University, Qingdao 266109, China; Engineering Laboratory of Genetic Improvement of Horticultural Crops of Shandong Province, Qingdao 266109, China; College of Horticulture, Qingdao Agricultural University, Qingdao 266109, China; Engineering Laboratory of Genetic Improvement of Horticultural Crops of Shandong Province, Qingdao 266109, China; College of Life Science, Qingdao Agricultural University, Qingdao 266109, China; College of Horticulture, Qingdao Agricultural University, Qingdao 266109, China; Engineering Laboratory of Genetic Improvement of Horticultural Crops of Shandong Province, Qingdao 266109, China; College of Horticulture, Qingdao Agricultural University, Qingdao 266109, China; Engineering Laboratory of Genetic Improvement of Horticultural Crops of Shandong Province, Qingdao 266109, China; College of Horticulture, Qingdao Agricultural University, Qingdao 266109, China; Engineering Laboratory of Genetic Improvement of Horticultural Crops of Shandong Province, Qingdao 266109, China

## Abstract

Skin color is one of the major traits of fruit appearance quality in pear (*Pyrus*) that affects the fruit commodity value. Russet skin protects pear fruits from environmental stresses and its formation process is closely linked to lignin accumulation. However, the molecular regulatory networks underlying russet skin formation in pear fruits involve complex secondary metabolic pathways and remain elusive. Here, we explored the regulatory mechanisms underlying lignin accumulation in pear skin based on transcriptome sequencing, co-expression network analysis, and gene expression profiling. We identified a WRKY transcription factor gene, *PbWRKY24*, that regulates russet skin formation in pear fruits. The relative expression of *PbWRKY24* in russet pear skin was significantly correlated with lignin content. We then verified the function of *PbWRKY24* in lignin accumulation via genetic transformation. DNA affinity purification sequencing revealed that PbWRKY24 directly binds to the promoter of a lignin biosynthesis gene, *PbPRX4*. This binding was confirmed by yeast one-hybrid, dual-luciferase, and electrophoretic mobility shift assays. Overexpression of *PbPRX4* in pear skin stimulated lignin accumulation and consequently promoted russet skin formation. This study provides a glimpse into the intricate lignin biosynthesis mechanisms during russet skin formation in pear fruits, which is of practical significance to pear breeding for fruit quality.

## Introduction

Pear (*Pyrus*) is one of the popular fruit crops worldwide producing nutritious fruits. Consumer demand for pear is continuously growing, which calls for enhancement of fruit quality and yield. Skin pigmentation is a major fruit trait that determines the market value and consumer acceptance of pear. Based on their skin color, pear fruits are divided into red, russet, and green groups [1]. The russet skin protects pear fruits from environmental stresses and affects fruit quality [2]. The formation of russet fruit skin is closely linked to lignin accumulation in pear [3]. Lignin contributes to the hydrophobic properties and wall rigidity of plant cells, enhancing defense against pathogens and insect pests [4, 5]. The key genes specific to lignin biosynthesis are shown to participate in russet skin formation in pear [6, 7], but the molecular mechanisms controlling lignin biosynthesis in russet pear skin are largely unknown.

**Figure 1:**
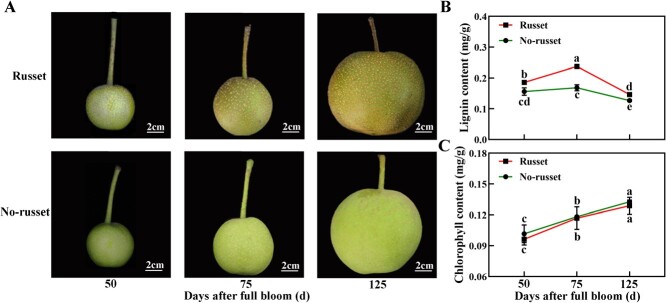
Changes in skin color and pigment contents of pear fruits. (**A**) Color development, (**B**) Lignin content, and (**C**) Chlorophyll content in fruit skin from 50 to 125 DAFB. Bars represent standard error of the mean (*n* = 4). Different lower case letters indicate significant differences by Tukey’s multiple range test (*P* <0 .05).

The complex composition of lignin in many plant species has been well documented. Lignin biosynthesis involves two steps, phenylpropanoid metabolism and lignin polymerization [8]. Pear shares a similar lignin biosynthesis pathway to other plant species previously reported. This pathway is regulated by sophisticated mechanisms involving rate-limiting and metabolic enzymes, as well as transcription factors (TFs) [9]. Many structural genes related to lignin biosynthesis contribute to russet pigmentation in pear skin: (i) genes encoding enzymes for monolignin biosynthesis, such as phenylalanine ammonialyase (PAL), 4-coumarate-coenzyme (CoA) ligase (4CL), cinnamoyl CoA reductase (CCR), cinnamyl alcohol dehydrogenase (CAD), shikimate/quinate hydroxycinnamoyl transferase (HCT), caffeic acid/5-hydroxyferulic acid O-methyltransferase (COMT), and coumarate 3-hydroxylase (C3H); and (ii) genes encoding enzymes for lignin polymerization, such as laccase (LAC) and peroxidase (PRX/POD/APX) [10, 11]. PRXs play an essential role in lignin biosynthesis in pear fruits, producing monomer phenoxy radicals and enabling lignin accumulation [7]. In particular, PtomtAPX/PRX is a crucial enzyme in lignin polymerization in the xylem of Chinese white poplar (*Populus tomentos* Carr.) [12]. Overexpression of *FaPOD/PRX* in transgenic strawberry (*Fragaria ananassa* Duch.) fruits results in increased lignin content and improved firmness [13].

Lignin biosynthesis is modulated *in vivo* by a set of TFs spanning multiple families [14]. WRKY, MYB, NAC, bZIP, and bHLH are predicted to be potential regulators of russet skin formation in pear [15]. The WRKY TF family members contain a unique DNA-binding site W-box (TTGACC/T) and a highly conserved WRKY domain [16, 17]. WRKY TFs upregulate the transcription of lignin biosynthesis-related genes by directly binding to their promoter region. For example, GhWRKY1-like binds to the promoters of *GhPAL6* and *GhCOMT1*, activating their expression and thus promoting lignin accumulation in cotton (*Gossypium hirsutum* cv. ‘YZ1’) [18]. DkWRKY8 and DkWRKY10 contribute to lignin accumulation in persimmon (*Diospyros kaki* Tunb.) through interacting with the promoter region of *DkCAD1* [19]. RhWRKY30 triggers *RhCAD1* expression to enhance lignin accumulation in rose (*Rosa* spp.) petals [20]. MdWRKY75e targets *MdLAC7* to regulate lignin biosynthesis in apple (*Malus domestica* Borkh.) [21]. Recently, a number of WRKY TFs have been predicted as putative regulators of fruit russeting in apple and pear [15, 22]. However, whether and how exactly WRKY TFs modulate lignin biosynthesis in the russet fruit skin of pear are still enigmatic.

Here, we found a WRKY TF, PbWRKY24, that was closely associated with lignin accumulation and russet pigmentation in pear fruit skin. PbWRKY24 enhanced russet skin pigmentation with increased lignin content by directly activating the transcription of lignin biosynthesis-related genes. PbWRKY24 was bound to the *PbPRX4* promoter, as revealed by DNA affinity purification sequencing (DAP-seq), yeast one-hybrid (Y1H), dual-luciferase (LUC), and electrophoretic mobility shift (EMSA) assays. This study uncovers a WRKY-type TF that contributes to russet pigmentation in pear fruit skin by regulating lignin biosynthesis. The results provide new clues to clarify the elaborate regulatory mechanisms of russet skin formation in pear and possibly other fruits.

## Results

### Color development in fruit skin of pear

By visual inspection, we observed that russet skin fruits turned russet during fruit development, whereas non-russet skin fruits remained unchanged in color (Fig. 1a). Russet skin fruits showed increased lignin content of pear skin across different stages of fruit development, when compared with non-russet skin fruits (Fig. 1b). The lignin content peaked at 0.23 mg/g in russet skin fruits 75 days after full bloom (DAFB), which was 1.41-fold higher than that of non-russet skin fruits. There was no significant difference in the chlorophyll content of pear skin between the two fruit types (Fig. 1c). The results indicated a close linkage between russet fruit skin and lignin accumulation in pear.

**Figure 2:**
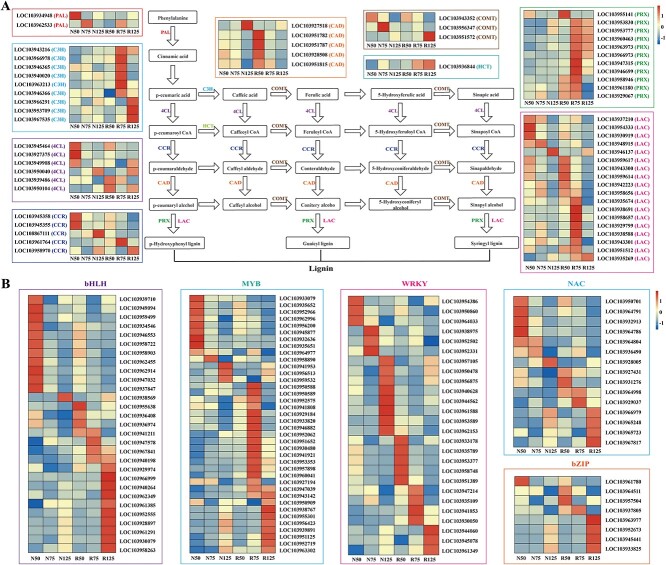
Simplified schemes and expression heat maps of lignin biosynthesis-related structural genes and TF family genes in pear skin. (**A**) Structural genes. (**B**) TF family genes. R/N50, 75, and 125: russet/non-russet skin fruits at 50, 75, and 125 DAFB. Detail information of those genes was listed in Table S4.

### Identification of differentially expressed genes

To decipher the molecular mechanisms behind the distinct phenotypes of pear fruits, RNA sequencing (RNA-seq) was employed to analyze skin samples of russet skin fruits (group R) and non-russet skin fruits (group N). The number of clean reads per library ranged from 19.90 to 27.54 million. Approximately 57.28%–78.06% of the reads were mapped to the *Pyrus bretschneideri* genome, with a uniquely mapped rate of 48.66%–70.76%. All biological replicates exhibited a strong correlation (Table S1, Table S2).

Differentially expressed genes (DEGs) were identified among samples at 50 (R50/N50), 75 (R75/N75), and 125 (R125/N125) DAFB groups. More DEGs were found between R75 versus R125 than between R50 versus R75, similar to the pattern in group N (Fig. S1a). Accordingly, the stage at 75–125 DAFB could be critical for russet pigmentation in pear fruit skin. Moreover, DEGs were analyzed between and among different sample groups (Fig. S1b). The number of DEGs between R versus N slightly increased following fruit development. To validate the RNA-seq transcriptome results, 12 DEGs were selected for measurement of transcript levels by quantitative real-time PCR. These genes were *PbPRX*, *PbCCR*, *PbC3H*, *Pb4CL*, *PbPAL*, *PbCAD*, and *PbHCT*, involved in lignin biosynthesis; and *PbWRKY24*, *PbMYB3*6, *PbNAC78*, *PbNAC100*, and *PbbZIP16*, which regulate lignin biosynthesis. The overall expression patterns of the selected DEGs showed high consistency (Fig. S2, Table S3), affirming the reliability of RNA-seq results.

We functionally categorized the DEGs and identified the biological pathways involved in russet skin formation. The DEGs between R75 and N75, as well as those between R125 and N125, were mostly enriched in pathways of ‘metabolic processes’, ‘cellular processes’, ‘single biological processes’, and ‘biological regulation processes’ (Fig. S3a, b). The DEGs between R75 and N75 were notably enriched in pathways of ‘phenylpropanoid biosynthesis’, ‘plant–pathogen interaction’, and ‘plant hormone signal transduction’ (Fig. S3c). The DEGs between R125 and N125 were principally enriched in pathways of ‘plant–pathogen interaction’, ‘plant hormone signal transduction’, ‘MAPK signaling pathway–plant’, and ‘phenylpropanoid biosynthesis’ (Fig. S3d). These results provided evidence for the potentially important role of these biological pathways in pear color development.

### Expression of lignin biosynthesis-related genes and transcription factors

To ascertain the effect of lignin biosynthesis on russet pigmentation in pear fruit skin, we looked at the expression patterns of lignin biosynthesis-related structural genes between groups N and R (Fig. 2a, Table S4). The expression levels of two *PALs* peaked in N50 and N75, respectively. Six *C3Hs* exhibited their highest expression levels in R75, and another three *C3Hs* peaked in R125. Expression of two *4CLs* peaked in R50, whereas another five *4CLs* were highly expressed in group N. Three *CCR*s showed their highest expression levels in N50 or N125, with another two *CCRs* peaking in R75 and R125, respectively. One *CAD* exhibited higher expression in N125, whereas another four *CADs* peaked in R50. Among *COMTs*, expression of LOC103943352 peaked in N50, and LOC103956347 was highly expressed in N75, whereas LOC103951572 reached its peak level in R125. *HCT* was expressed at low levels in the N group, with increased levels in the R group and being highest in R125. High expression levels of *PRXs* were found in the R group compared with the N group; LOC103955141 peaked in R50 and another 10 *PRXs* peaked in R75. Besides, 18 *LAC* genes were identified, five of which reached their highest expression levels in the N group and the other 13 increased to varying degrees in the R group. These genes may be crucial for lignin biosynthesis during russet skin formation in pear.

In addition to the structural genes, TFs also play crucial roles in lignin accumulation. To reveal the transcriptional regulatory network of russet pear skin, 116 putative TF genes, including those encoding MYBs, WRKYs, NACs, bZIPs, and bHLHs, were identified. The expression profiles of these TF genes exhibited highly dynamic changes during the formation of pear russet skin (Fig. 2b, Table S4). Some TF genes showed higher expression levels in the group N compared with the group R. This was exemplified by significant upregulation of 11 *bHLHs*, 9 *MYBs*, 3 *WRKYs*, 5 *NACs*, and 1 *bZIPs* in N50. Additionally, one *MYB*, three *WRKYs*, and one *NAC* peaked in N75. One *bHLH*, three *MYBs*, eight *WRKYs*, and one *NAC* reached their highest levels in N125. In contrast, a set of TFs were highly expressed during russet formation in russet skin fruits, indicating their positive role in regulating russet formation. For example, four *bHLHs*, five *WRKYs*, two *NAC*s, and two *bZIPs* were significantly upregulated in R50. Additionally, 10 *bHLHs*, 9 *MYBs*, 3 *WRKYs*, 4 *NACs*, and 4 *bZIPs* showed their highest levels in R125. Notably, 4 *bHLHs*, 16 *MYBs*, 4 *WRKYs*, 2 *NACs*, and 1 *bZIP* were upregulated in group R, with expression levels peaking in R75, similar to the pattern of lignin content in russet skin fruits (Fig. 2b, Table S4).

### Identification of lignin-related co-expression network modules

**Figure 3:**
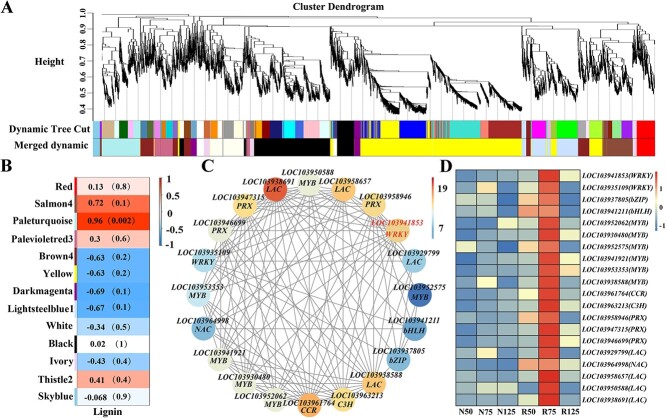
WGCNA of DEGs identified in pear skin. (**A**) Hierarchical cluster tree displaying 13 modules of co-expressed genes. Tree leaves and branches represent the DEGs and modules, respectively. (**B**) Trait correlations and corresponding *P*-values for the 13 modules. (**C**) Cytoscape representation of co-expressed genes in the ‘Paleturquoise’ module. Member gene IDs and common names are given. (**D**) Heat map showing the expression patterns of co-expressed genes in the ‘Paleturquoise’ module. Detailed information of DEGs in the ‘Paleturquoise’ module is listed in Table S5.

To uncover the molecular regulatory network of lignin biosynthesis, weighted gene co-expression network analysis (WGCNA) was performed on the DEGs, leading to identification of 13 modules (Fig. 3a, b). Module–trait correlation result revealed that the ‘Paleturquoise’ was closely related to lignin content (*r* =0 .96, *P* =0 .002) (Fig. 3b). Thus, the genes in this module may play pivotal roles in lignin accumulation, contributing to russet skin formation in pear fruits. The DEGs in the ‘Paleturquoise’ module were primarily enriched in pathways of ‘plant hormone signal transduction’, and ‘phenylpropanoid biosynthesis’ (Fig. S4b), similar to observations between R75 and N75 and between R125 and N125 (Fig. S3c, d).

A total of 270 genes were identified in the ‘Paleturquoise’ module (Table S5). Among these genes, nine structural genes were found involved in lignin biosynthesis—*LOC103961764* (*CCR*), *LOC103963213* (*C3H*), *LOC103929799* (*LAC*), *LOC103958657* (*LAC*), *LOC103938691* (*LAC*), *LOC103938588* (*LAC*), *LOC103958946* (*PRX*), *LOC103947315* (*PRX*), and *LOC103946699* (*PRX*). Additionally, there were 11 TF genes involved in lignin biosynthesis and transportation—*LOC103941853* (*WRKY*), *LOC103935109* (*WRKY*), *LOC103941921* (*MYB*), *LOC103952062* (*MYB*), *LOC103953353* (*MYB*), *LOC103952575*(*MYB*), *LOC103950588* (*MYB*), *LOC103930480* (*MYB*), *LOC103937805* (*bZIP*), *LOC103941211* (*bHLH*), and *LOC103964998* (*NAC*).

To visualize the relationship between the TF genes and lignin biosynthesis-related genes, we conducted network diagram analysis on the 20 genes in the ‘Paleturquoise’ module. Among the 11 TF genes, *LOC103941853* (*WRKY*) had the largest number of edges, 14 (Fig. 3c). The transcription levels of the 20 genes were generally consistent (Fig. 3d, Table S5). These results indicated that LOC103941853 (WRKY) is a key TF potentially contributing to russet skin formation in pear fruits through modulation of lignin accumulation.

### *PbWRKY24* modulates russet skin pigmentation in pear fruit

**Figure 4:**
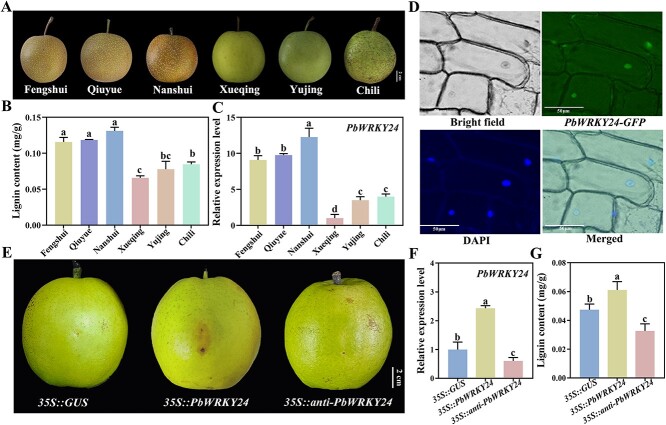
Subcellular localization and gene expression of *PbWRKY24* in pear fruit skin. (A) Color phenotypes, (**B**) lignin content, and (**C**) *PbWRKY24* expression in pear skin of six different cultivars. (**D**) Microphotographs showing *PbWRKY24-GFP* fusion plasmid transient expression in epidermal cells of onion. (**E**) Skin phenotype, (**F**) *PbWRKY24* expression, and (**G**) lignin content in fruits upon overexpression and silencing of the *PbWRKY24* gene.

To identify the relationship of LOC103941853 in pear and WRKYs in *Arabidopsis*, we constructed a phylogenetic tree based on amino acid sequence alignments of 61 AtWRKYs. LOC103941853 was most closely related to AtWRKY24 (Fig. S5). Therefore, LOC103941853 was designated PbWRKY24. Additionally, there were higher *PbWRKY24* transcription levels and lignin contents in russet skin cultivars (‘Qiuyue’, ‘Nanshui’, and ‘Fengshui’) than in non-russet skin cultivars (‘Xueqing’, ‘Chili’, and ‘Yujing’) (Fig. 4a–c). Then, we transiently expressed *PbWRKY24-GFP* in epidermal cells of onion. Subcellular location assay revealed that the PbWRKY24-GFP fusion protein was localized to the nucleus (Fig. 4d).

Next, we investigated the role of *PbWRKY24* in the regulation of russet skin formation in pear. Three plasmids—*35S::GUS* (control), *35S::PbWRKY24*, and *35S::anti-PbWRKY24*—were constructed and injected into pear skin by agroinfiltration. *PbWRKY24-*overexpressing fruits showed enhanced russet pigmentation around the injection site, whereas the control and *PbWRKY24-*silenced fruits basically remained unchanged in color (Fig. 4e). The *PbWRKY24* transcription level in the skin of *PbWRKY24-*overexpressing fruits was 2.43- and 4.05-fold higher than that of the control and *PbWRKY24-*silenced fruits, respectively (Fig. 4f). This pattern corresponded to the changes in lignin content, which peaked in pear skin of *PbWRKY24-*overexpressing fruits, 28.99% and 86.92% greater than that of the control and *PbWRKY24-*silenced fruits, respectively (Fig. 4g).

Furthermore, we determined whether PbWRKY24 participates in the transcriptional regulation of lignin biosynthesis-related genes, represented by the nine structural genes in the ‘Paleturquoise’ module. Compared with *PbWRKY24-*silenced fruits, *PbPRX4*, *PbLAC7*, *PbLAC4–1*, *PbC3H*, and *PbCCR* were 7.78-, 0.25-, 0.29-, 2.96-, and 0.22-fold upregulated, respectively, in pear skin of *PbWRKY24-*overexpressing fruits. The opposite was true for *PbLAC12* and *PbPRX11–1*, which were 1.48- and 0.50-fold downregulated in pear skin of *PbWRKY24-*overexpressing fruits*.* The expression levels of *PbPRX11* and *PbLAC*4 in pear skin showed no significant difference between *PbWRKY24-*overexpressing and *-*silenced fruits (Fig. S6). Notably, the expression patterns of *PbPRX4*, *PbC3H*, and *PbCCR* were consistent with the trend of *PbWRKY24* in the fruit skin of transgenic pear (Fig. 4g, Fig. S6).

### PbWRKY24 enhances lignin accumulation by directly binding to *PbPRX4* promoter

To elucidate the mechanisms underlying *PbWRKY24-*mediated transcriptional regulation of lignin accumulation in russet fruit skin, we mapped the DNA-binding sites of PbWRKY24 across the *P. bretschneideri* genome using DNA purification and sequencing (DAP-seq). There were 12 830 highly reliable PbWRKY24-binding sites. Most of the binding peaks of PbWRKY24 (60.61%) were distributed in the promoter and intergenic regions, with 12.78% in the exon region and 26.61% in the intron region (Fig. S7a). Venn diagram shows the number of unique and shared PbWRKY24-binding sites between two replicate experiments (Fig. S7b). The PbWRKY24-binding peaks were distributed on different chromosomes (Fig. S7c). Gene Ontology (GO) analysis revealed that the candidate target genes of PbWRKY24 were mainly enriched in terms of ‘cellular metabolic process’, ‘organic substance metabolic process’, and ‘system development’ (Fig. S7d). Kyoto Encyclopedia of Genes and Genomes (KEGG) analysis showed that the candidate target genes were mainly enriched in pathways of ‘spliceosome’, ‘nucleocytoplasmic’, and ‘ribosome biogenesis in eukaryotes transport’ (Fig. S7e).

**Figure 5:**
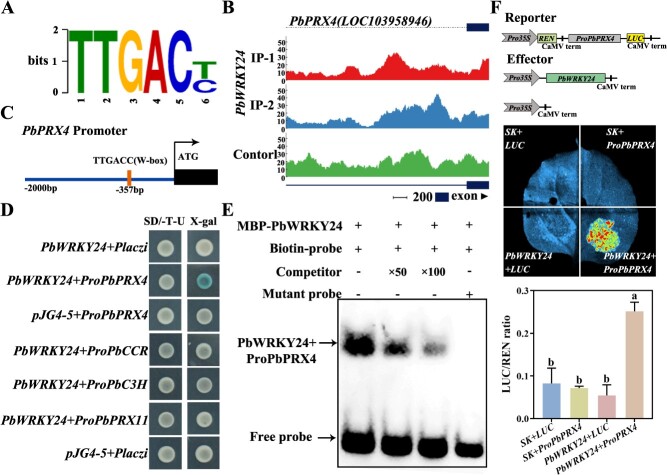
PbWRKY24 directly binds to the promoter of *PbPRX4* and thereby enhances its transcription. (**A**) DNA-binding sites of PbWRKY24. (**B**) Visualization and analysis of *PbPRX4* as a potential target gene of PbWRKY24. (**C**) Schematic diagram illustrating the distribution of W-box in the *PbPRX4* promoter. (**D**) Y1H assay showing that PbWRKY24 binds to the *PbPRX4* promoter. (**E**) EMSA assay showing that PbWRKY24 binds to the *PbPRX4* promoter. (**F**) LUC activity assay showing that PbWRKY24 activates the *PbPRX4* promoter. Data analysis of the relative firefly luciferase/*Renilla* luciferase (LUC/*R*EN) ratio from transient expression assays in the figure.

A total of 156 potential target genes were identified by overlapping analysis of DEGs between the ‘Paleturquoise’ module and DAP-seq data (Fig. S8a). Using the MEME suite and visualization analysis, we identified the motif sequence with the most significant enrichment of PbWRKY24-binding sites—‘TTGACC/T’, a typical W-box element (Fig. 5a, b, Fig. S8c). Two W-box elements were found in the promoter region of *PbC3H*, and one in each of the *PbPRX4*, *PbCCR*, and *PbPRX11* promoters (Fig. 5c, Fig. S8b). These results suggested that *PbC3H*, *PbCCR*, *PbPRX11*, and *PbPRX4* are the target genes regulated by PbWRKY24.

We then asked whether PbWRKY24 directly regulates the transcription of *PbC3H*, *PbCCR*, *PbPRX11*, and *PbPRX4* in pear skin. Y1H analysis showed that PbWRKY24 directly binds to the *PbPRX4* promoter (Fig. 5d). In EMSA, PbWRKY24-MBP fusion protein was bound to the *PbPRX4* promoter probe *in vitro*, and the extent of this binding was reduced by the addition of unlabeled competitor probe (Fig. 5e). Further, we conducted LUC activation assay to confirm the binding interaction. The activity of the *PbPRX4* promoter was enhanced in 4-week-old leaf epidermal cells of tobacco upon co-transformation with the effector vector *Pro35S:PbWRKY24* and the reporter vector *ProPbPRX4:LUC* (Fig. 5f). The results provided robust evidence that PbWRKY24 activates the transcription of *PbPRX4* by directly binding to its promoter.

### *PbWRKY24* overexpression enhances lignin accumulation in transgenic tobacco

**Figure 6:**
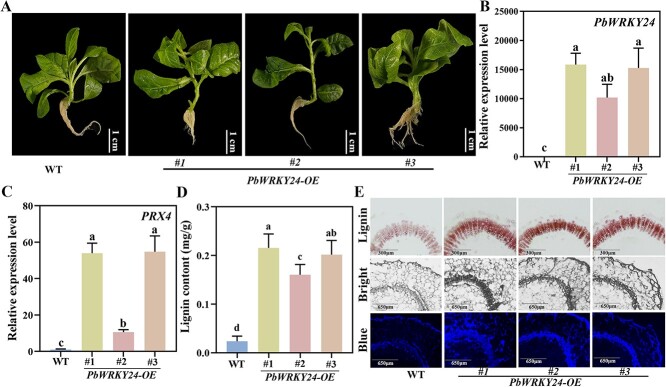
Functional identification *PbWRKY24* in transgenic tobacco plants. (**A**) Phenotype, (**B**) *PbWRKY24* expression, (**C**) *PRX4* expression, (**D**) lignin content, and (**E**) lignin staining and autofluorescence in stems of WT and *PbWRKY24-*overexpressing (*PbWRKY24-OE*) lines.

To validate the function of *PbWRKY24* in lignin biosynthesis, we constructed transgenic tobacco plants of *35S::PbWRKY24* using an *Agrobacterium*-mediated transformation system (Fig. 6a). The transcription levels of *PbWRKY24* and *PRX4* in transgenic plants were 13 776.26- and 39.81-fold higher than those of wild-type (WT) plants, respectively (Fig. 6b, c). Western blot assay was used to confirm the transgenic lines (Fig. S9). The lignin content of transgenic plants was 5.80- to 8.14-fold greater than that of WT plants (Fig. 6d). The level of lignin staining in tobacco stems was higher in *PbWRKY24*-overexpressing plants than that in empty-vector plants (Fig. 6e). The autofluorescence within the stem sections of *PbWRKY24*-overexpressing plants was also more pronounced compared with the control plants (Fig. 6e). The results confirmed that PbWRKY24 enhances lignin biosynthesis in russet pear skin by activating *PbPRX4* transcription.

### *PbPRX4* overexpression promotes lignin accumulation and russet skin formation in pear

**Figure 7:**
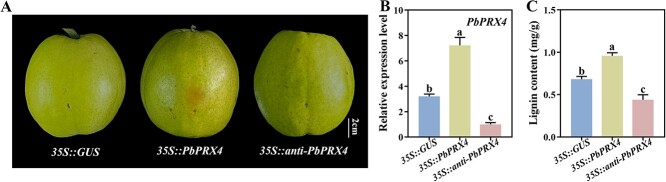
Overexpression and silencing of the *PbPRX4* gene in pear fruit skin. (**A**) Skin phenotype. (**B**) *PbPRX4* expression. (**C**) Lignin content.

To verify whether *PbPRX4* is also involved in lignin accumulation and russet skin formation in pear, we injected *35S::GUS* (control), *35S::PbPRX4*, and *35S::anti-PbPRX4* injected into pear skin by agroinfiltration. *PbPRX4*-overexpressing fruits showed a strong russet phenotype at the injection site (Fig. 7a), corresponding to higher *PbPRX4* transcription level (by 2.25-fold; Fig. 7b) and lignin content (by 1.41-fold; Fig. 7c) than the controls. No color change occurred in the other two groups, with lower *PbPRX4* expression level and lignin content in pear skin of *PbPRX4*-silenced fruits than the controls (Fig. 7a–c). The results suggested that *PbPRX4* plays an active role in regulating lignin accumulation and russet skin formation in pear fruits.

## Discussion

**Figure 8:**
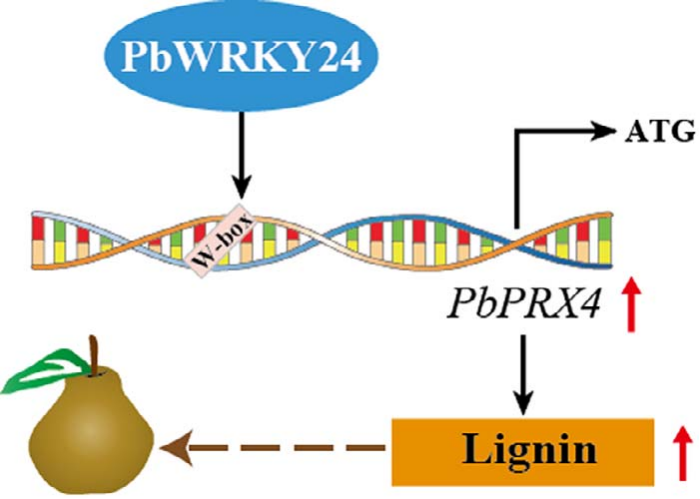
Conceptual model for PbWRKY24 regulation of lignin accumulation in russet pear skin. PbWRKY24 modulates lignin biosynthesis pathway in russet fruit skin of pear by directly binding to the *PbPRX4* promoter and activating *PbPRX4* transcription.

The physiological mechanisms related to russet skin in pear fruits have been characterized [23], but the molecular regulatory networks responsible for russet skin formation remain elusive. Here, we identified a key TF, PbWRKY24, that regulates russet skin formation in pear fruits. Based on the omics data, we developed a model explaining the molecular mechanisms by which PbWRKY24 enhances lignin accumulation in russet pear skin. PbWRKY24 promotes lignin biosynthesis by activating the *PbPRX4* promoter, which facilitates the formation of russet pear skin (Fig. 8).

Several studies reported higher lignin content in russet skin than in green skin of pear and apple fruits [24, 25]. Consistently, we observed that the content of lignin in the russet skin of pear fruits increased compared with that of green pear skin (Fig. 1a, b). KEGG analysis revealed that the ‘phenylpropanoid biosynthesis’ pathway was enriched for the DEGs between russet and non-russet skin fruits at 75 and 125 DAFB (Fig. S3). This indicates that lignin biosynthesis is a major process contributing to russet skin formation in pear. Many researchers, including Heng et al. (2014), Wang et al. (2014), and Wu et al. (2023), have used pear cultivars and their mutants to explore different skin pigmentation patterns [6, 15, 25]. These results indicated that phenylpropanoid metabolism is involved in the russet formation process, and fruit skin is likely to be regulated by lignin biosynthesis, consistent with our findings. However, the lignin content of russet skin fruits at 125 DAFB was lower than that of 50 DAFB fruits (Fig. 1a). The possible reason is that the formation of russet fruit skin is related not only to the phenylpropanoid biosynthesis pathway but also to the lipid biosynthesis pathway [10].

Furthermore, several studies have reported the putative molecular regulatory pathways enriched for DEGs that are associated with the formation of russet skin fruits [15, 24]. For example, genes involved in lignin biosynthesis, such as *PRX*, *COMT*, *C4H*, and *LAC*, are highly expressed in the russet skin of apple and pear fruits [15, 24]. We detected higher expression levels of DEGs encoding key enzymes for lignin biosynthesis (*4CLs*, *C3Hs*, *CCRs*, *CADs*, *COMTs*, *HCT*, *LAC*s, and *PRX*s) in pear fruits. In most cases, the transcription patterns of these DEGs corresponded to the dynamics of lignin biosynthesis in russet skin fruits (Fig. 1b, 2a). This led us to posit that these highly expressed lignin biosynthesis-related genes play critical roles in russet skin formation in pear.

Some TFs are well known to activate or inhibit the transcription of associated enzyme genes at post-transcriptional and transcriptional levels, leading to dynamic changes in lignin biosynthesis [26]. Among them, WRKY TFs contribute to the maintenance of normal plant development by up- or downregulating lignification [16], but whether they regulate lignin biosynthesis in russet pear skin has rarely been reported. Based on WGCNA analysis, we established a high correlation between the ‘Paleturquoise’ module and the lignin content of pear skin (Fig. 3b). Twenty lignin biosynthesis-related genes in this module were likely to be responsible for russet skin formation in pear fruits after full bloom, as indicated by their higher transcription levels in russet skin fruits than in non-russet skin fruits (Fig. 3d). Among the TF genes, *PbWRKY24* was most closely associated with other genes in the ‘Paleturquoise’ module (Fig. 3c). Moreover, there were higher *PbWRKY24* transcription levels in russet skin cultivars than in non-russet skin cultivars (Fig. 4a–c). These results indicate that PbWRKY24 is a putative positive regulator in the formation of russet pear skin [22].

Indeed, fruits overexpressing *PbWRKY24* showed enhanced russet pigmentation, with increased lignin content in pear skin (Fig. 4e, f). Overexpression of *PbWRKY24* in tobacco also stimulated lignin accumulation (Fig. 6d, e). These results underscore the critical role that *PbWRKY24* plays in lignin accumulation and russet pigmentation of pear skin. WRKY TFs take part in signal transduction networks regulating biotic/abiotic stresses, growth, and development in woody plants [27]. Whether PbWRKY24 performs similar functions in pear still needs to be verified. While the present study only identified *PbWRKY24*, other TFs may interact with this TF to modulate lignin accumulation in russet pear skin. For example, a set of TF genes, including *MYBs*, *WRKYs*, *NACs*, *bHLHs*, and *bZIPs* were highly expressed in russet pear skin (Fig. 2b, Fig. 3c). These collective results provide novel insight into the transcriptional regulation of lignin biosynthesis in russet pear skin.

WRKY TFs have been demonstrated to enhance lignin accumulation in plants by directly binding to the W-box motif in the promoter regions of *PAL*, *CAD*, and *COMT* genes [17–19]. In our study, similar to *PbWRKY24*, three lignin-related structural genes—*PbPRX4*, *PbC3H*, and *PbCCR*—were upregulated in pear skin by overexpression of *PbWRKY24* and downregulated by silencing of *PbWRKY24* (Fig. 4g, Fig. S6). DAP-seq results showed that the downstream gene *PbPRX4* was directly regulated through the binding of PbWRKY24 to the W-box element in its promoter region (Fig. 5a, c), as verified by Y1H, EMSA, and LUC assays (Fig. 5d–f). Multiple *PRX* genes have functional diversity, some of which play important roles in lignin accumulation in russet pear skin [12, 13]. We found that 11 *PRXs* were upregulated in the russet skin of pear fruits (Fig. 2a), although their different trends indicate potential functional diversity. A potential regulatory relationship was established between *PbWRKY24* and *PbPRX4* based on their strongly correlated transcription patterns in the russet skin fruits and *PbWRKY24-*overexpressing fruits (Fig. 4g, Fig. S6).

In soybean (*Glycine max* (L.) Merr.), GmWRKY172 upregulates the expression of *PRXs*, thereby increasing lignin accumulation [28]. Our results indicated that PbWRKY24 increased lignin content and promoted russet skin formation in pear fruits by directly activating *PbPRX4* (Fig. 4e, f, Fig. 6, and Fig. S6). PRXs additionally have a direct influence on lignin biosynthesis by regulating the terminal reaction in the lignin biosynthesis pathway [29]. Fernández-Pérez et al. (2015) validated the function of *AtPRX4* in lignin biosynthesis in *Arabidopsis* using a knockout mutant of *AtPRX4*, the closest homolog to PbPRX4 [30]*.* We observed that the lignin content of pear skin was increased upon overexpression of *PbPRX4*, along with enhanced russet pigmentation (Fig. 7). Accordingly, *PbPRX4* is a major gene in the lignin biosynthesis pathway contributing to russet skin formation in pear fruits. However, PbWRKY24 binds to the promoter of *PbPRX4* only, but not other lignin biosynthesis-related structural genes (Fig. 5d). There may be another mechanism by which PbWRKY24 interacts with other TFs or genes to regulate lignin biosynthesis in pear fruit skin. Therefore, lignin accumulation in russet pear skin is probably controlled by a multilevel regulatory network that requires further investigation.

## Conclusions

This study uncovered the molecular mechanisms for the formation of russet pear skin based on transcriptome sequencing, co-expression network analysis, and gene expression profiling. We identified a WRKY TF gene, *PbWRKY24*, which is closely linked to russet skin formation in pear fruits. Overexpression of *PbWRKY24* stimulated lignin accumulation in pear skin and tobacco plants. *PbPRX4* is a major gene in the lignin biosynthesis pathway in pear skin, and its promoter can be transcriptionally activated by directly binding to PbWRKY24. Overexpression of *PbPRX4* in pear skin increased lignin content and enhanced russet pigmentation. This research sheds light on the regulatory networks controlling russet skin formation in fruits and provides a gene resource for the enhancement of pear fruit quality.

## Materials and methods

### Plant materials

A pear F_1_ population derived from a cross between *Pyrus pyrifolia* cv. ‘Niitaka’ (russet skin cultivar) and *P. bretschneideri* cv. ‘Dangshansu’ (non-russet skin cultivar) was used in this study. Trees (17 years old) were planted at a density of 2.5 × 0.5 m at the Fruit Research Station of Qingdao Agricultural University (Laiyang, Shandong Province, China). Fruit samples were randomly taken from the southern side of tree canopy between 8 and 10 a.m. at 50, 75, and 125 DAFB. At each sampling time, four biological replicates were harvested, with at least four fruits collected from two trees per replicate. Mature ‘Qiuyue’, ‘Nanshui’, ‘Fengshui’, ‘Xueqing’, ‘Chili’, and ‘Yujing’ fruits were harvested at Yantai Academy of Agricultural Sciences (Yantai, Shandong Province, China). Fruit skin samples were collected with a peeler, snap-frozen in liquid nitrogen, and stored at −80°C until used for pigment measurement, transcriptome sequencing, and gene expression analysis. Mature ‘Korla’ fruits characterized by thin skin were used for transgenic infection. Tobacco (*Nicotiana benthamiana* L.) was used for LUC assay and transgenic transformation.

### Lignin and chlorophyll analysis

Total lignin was extracted and was performed as described previously [31]. Tissue samples (0.5 g) were homogenized in 5 ml of 80% acetone. After centrifugation (13 000 × *g*, 20 min), the absorbance of the supernatants was measured at 280 nm using a UV-2550 ultraviolet spectrophotometer (Shimadzu Corp., Kyoto, Japan). Measurement of total chlorophyll content was performed after sample extraction with 80% acetone [31]. Absorbance of the supernatant was measured at 663 and 645 nm using a UV-2550 ultraviolet spectrophotometer. Four independent biological replicates were performed for each experiment.

### RNA sequencing and data processing

Total RNA extraction from fruit skin samples was accomplished using the RNAprep Pure Plant Kit (Tiangen, Beijing, China). RNA samples were reverse transcribed using the SPARKscript II RT Plus Kit (SparkJade, Jinan, China). The complementary DNA fragments were purified as described previously [31]. The resulting library was sequenced at Gene Denovo Biotechnology Co., Ltd. (Guangzhou, Guangdong Province, China). In total, six sets of raw reads were obtained at 50, 75, and 125 DAFB, with three sets in group R (R50, R75, and R125) and three sets in group N (N50, N75, and N125). Pearson’s correlation coefficients were calculated for three biological replicates using the log_10_-transformed fragments per kilobase of transcript per million mapped reads (FPKM) method [32].

The raw reads were processed using BMKCloud (http://www.biocloud.net) and filtered as described by Ma et al [33]. The clean reads were mapped onto the *P. bretschneideri* genome (https://www.ncbi.nlm.nih.gov/datasets/genome/GCF_000315295.1/) using SOA-Paligner/soap2 [34]. Clean reads were then aligned with the reference genome and transcripts were reconstructed using Cufflinks [35].

### Identification of differentially expressed genes

The Hisat2 tool was used to map the clean reads to the reference genome [36], followed by normalization into FPKM reads as previously described [32]. The original *P*-values were adjusted using the Benjamini and Hochberg [37] approach to minimize the false discovery rate (FDR). DEGs with |fold change| ≥2 and FDR <0.05 were considered significant.

### Functional annotation of differentially expressed genes

Functional and pathway enrichment analyses of DEGs were respectively conducted using the GO database [38] and KEGG database [39]. Hypergeometric tests were performed to determine significant GO and KEGG enrichment in comparison to the genomic background. The *P*-values were corrected using FDR with a threshold ≤0.05.

### Construction of co-expression network

The R ‘WGCNA’ package (version 1.72–5) was used to perform co-expression network analysis [40]. WGCNA was constructed using the following parameters: gene expression threshold = 1, module similarity threshold = 0.25, and minimum gene number of modules = 30. Module eigengene values were calculated to the correlations between modules and lignin content in 18 samples. Cytoscape (version 3.10.3) was used to construct and visualize the co-expression network of genes in the module ‘Paleturquoise’.

### Quantitative real-time PCR assay

Quantitative real-time PCR (qPCR) assay was performed using LightCycler R480 SYBR Green Master (Roche, Mannheim, Germany) [33]. Specific primers (Supplementary Table S6) were designed using Primer 5 [41], and *Actin* (GenBank: AB190176) served as a reference gene. Gene expression levels were analyzed using the 2^–ΔΔCT^ method [42].

### Subcellular localization of PbWRKY24

Subcellular localization assay was performed using the method of Wang et al [43]. The coding sequence (CDS) of *PbWRKY24* without the stop codon was inserted into the *pMDC83* vector (GFP expression vector), generating a *35S::PbWRKY24-GFP* construct. *35S::GFP* and *35S::PbWRKY24-GFP* were separately transformed into *Agrobacterium tumefaciens* strain GV3101 and then transfected into onion epidermal cells. The epidermal cells were observed at ×20 magnification by confocal microscopy (FV10-ASW, Olympus, Japan) 3 days after transfection.

### Vector construction and transformation in pear

The *PbWRKY24* CDS (without the stop codon) was cloned into the overexpression vector *pBI121* to generate the *35S::PbWRKY24* and *35S::anti-PbWRKY24* constructs, respectively. The primers are listed in Supplementary Table S6. *35S::PbWRKY24*, *35S::anti-PbWRKY24*, and *35S::GUS* (control) were transformed separately into *A. tumefaciens* strain EHA105. The infiltrated samples were incubated overnight in the dark at room temperature (28°C), then exposed to white light (540 μmol·m^−2^·s^−1^) with a 16-h photoperiod at 25°C in a growth chamber [31].

### Protein extraction and western blotting

Protein extraction and western blotting assays were performed as previously described [44]. The specific anti-PbWRKY24 antibody was customized by GeneCreate (Wuhan, Hubei Province, China). The anti-ACTIN antibody (PTM BioLabs Inc., Hangzhou, Zhejiang Province, China) was used as an internal reference.

### DNA sequencing and data analysis

DAP-seq was accomplished by Gene Denovo Biotechnology Co., Ltd (Guangzhou). The isolation of genomic DNA from fruit skin samples (0.5 g) was performed using a plant genomic DNA extraction kit (Tiangen, Beijing, China). The *PbWRKY24* CDS was inserted into the HaloTag vector (Gene Denovo Biotechnology Co., Ltd) to generate a *PbWRKY24*-HaloTag construct for protein expression. The expressed *PbWRKY24*-HaloTag fusion protein was purified and captured using Magne HaloTag Beads (Promega, Fitchburg, WI, USA). The *PbWRKY24*-HaloTap fusion protein was incubated with the DAP-seq DNA library. After PCR amplification by Kidio Biotech. (Guangzhou, Guangdong Province, China), the eluted DNAs were sequenced using Illumina HiSeqTM 4000 (Illumina Inc., San Diego, CA, USA). Magnetic beads lacking *PbWRKY24* were used to prepare DAP-seq DNA libraries as negative controls.

Bowtie2 was used to align DAP-seq reads with the *P. bretschneideri* genome [45]. MACS2 was adopted to identify read-enriched regions from DAP-seq data [46]. Regions with *q*-value <0.05 were detected as peaks, and peak-related genes were annotated using the R ‘DiffBind’ package (version 2.8). The MEME suit (http://meme-suite.org/) was used to annotate peaks [47].

### Y1H assay

Protein identification was performed by Y1H assay [48]. The *PbWRKY24* CDS was inserted into the *pJG4–5* vector. Each promoter fragment of potential target genes (*PbPRX4*, *PbPRX11*, *PbC3H*, and *PbCCR*) was inserted into the *pLacZi* vector. The primers used are listed in Supplementary Table S6.

### LUC assay

The effect of PbWRKY24 on *PbPRX4* was verified by LUC assay [48]. The *PbWRKY24* CDS was inserted into *pGreenII 62-SK* (an effector vector), and the promoter fragment of *PbPRX4* was inserted into *pGreenII 0800-LUC* (a reporter vector). The effector and reporter vectors were co-transformed into leaves of 4-week-old tobacco plants. The Infinite M200 (Tecan, Switzerland) was used to measure signals from firefly LUC and *Renilla* LUC. The primers used for LUC assay are provided in Supplementary Table S6.

### EMSA

The *PbWRKY24* CDS was cloned into the vector pMCSG19 (tagged with maltose-binding protein) and then transformed into *Escherichia coli* strain BL21 (Weidibio, Shanghai, China) for PbWRKY24 protein expression. The *PbPRX4* promoter fragments were constructed using biotin-labeled oligonucleotides, unlabeled oligonucleotides (as a competitor), or a mutant probe (Invitrogen, Shanghai, China). The primers are listed in Table S6. The EMSA was conducted using a LightShift chemiluminescence EMSA kit (Thermo Fisher Scientific, Waltham, MA, USA) as described by Dang et al. (2021) [48].

### Genetic transformation in tobacco

The genetic transformation of tobacco was carried out [49]. The *PbWRKY24* CDS was cloned into the *pBI121* vector to generate *35S::PbWRKY24*. The construct was transformed into tobacco leaves using EHA105 *Agrobacterium-*competent cells. The primers used are listed in Supplementary Table S6.

### Wiesner staining and microscopy

The Wiesner reaction (phloroglucinol-HCl) was performed to visualize lignification according to the method described by Li et al. (2019) [50]. Plant stems were sliced by a razor blade and then dyed in Weisner reagent for 10 min. An EVOS smart fluorescence microscope (Thermo Fisher Scientific) was used to observe autofluorescence within the stem sections.

### Statistical analysis

Each experiment was independently repeated at least three times. Data represent the means ± SD of at least triplicate experiments. Analysis of variance (ANOVA) followed by Tukey’s multiple range test was used to test the overall significance of differences among treatments (*P* <0 .05). All data were analyzed in SPSS v22.0 (IBM Corp., Armonk, NY, USA).

## Supplementary Material

Web_Material_uhae300

## Data Availability

All data supporting the findings are available within the paper or from the corresponding author upon request. The RNA sequencing data have been deposited in the National Center for Biotechnology Information (NCBI) SRA database under accession number PRJNA948064.
