# Supplementary material for: A transcription factor, PbWRKY24, contributes to russet skin formation in pear fruits by modulating lignin accumulation
Source: Hortic Res. 2024 Oct 18;12(2):uhae300. doi: 10.1093/hr/uhae300 (PMC11822408; doi:10.1093/hr/uhae300)
Supplement: Web_Material_uhae300 [file web_material_uhae300.zip › Suppiementary Figures 9.22.docx]

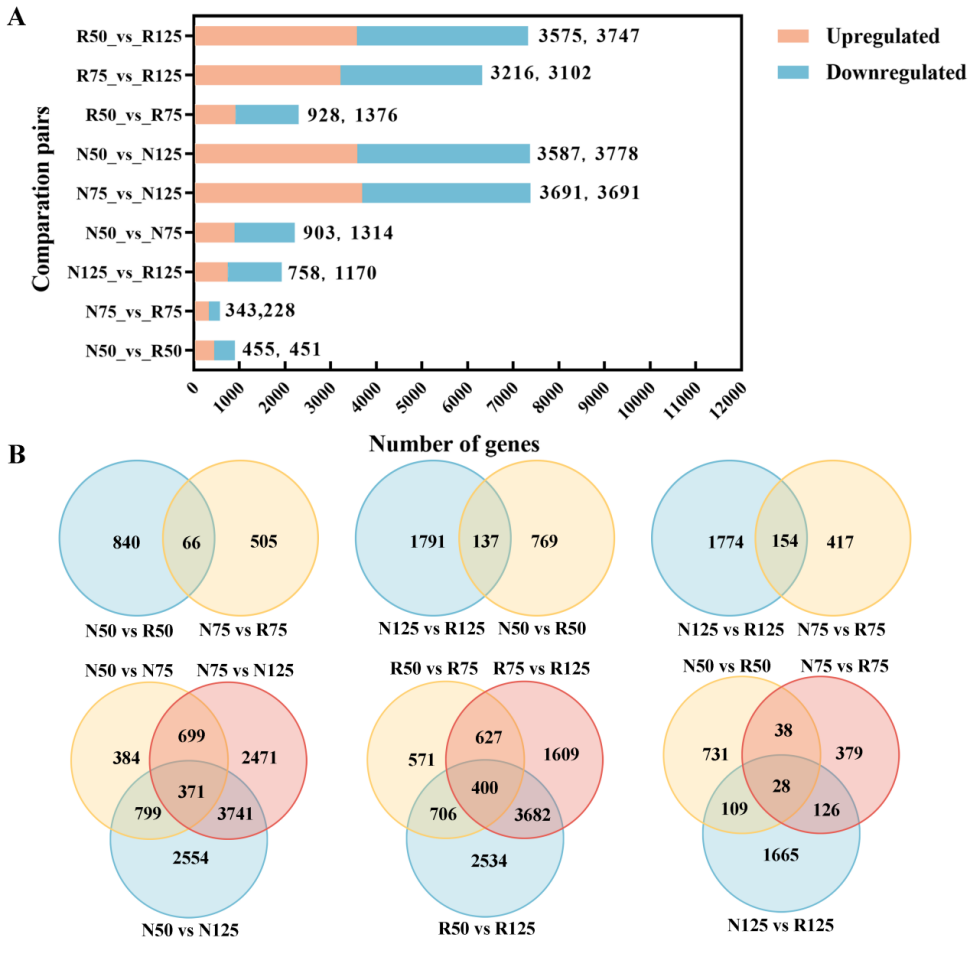


**Figure S1.** Differentially expressed genes (DEGs) identified by RNA sequencing in pear skin of russet (R) and non-russet skin (N) fruits at various developmental stages. (**A**) Numbers of DEGs between stages in each group and between groups at each stage. (**B**) Venn diagrams showing the unique and shared DEGs between groups and among groups at various stages. False discovery rate < 0.05 and |log_2_ fold change| > 1 were used as cut-off criteria for significance. R50, R75, and R125: russet skin fruits at 50, 75, 125 days after full bloom, respectively; N50, N75, and N125: non-russet skin fruits at 50, 75, 125 days after full bloom, respectively.


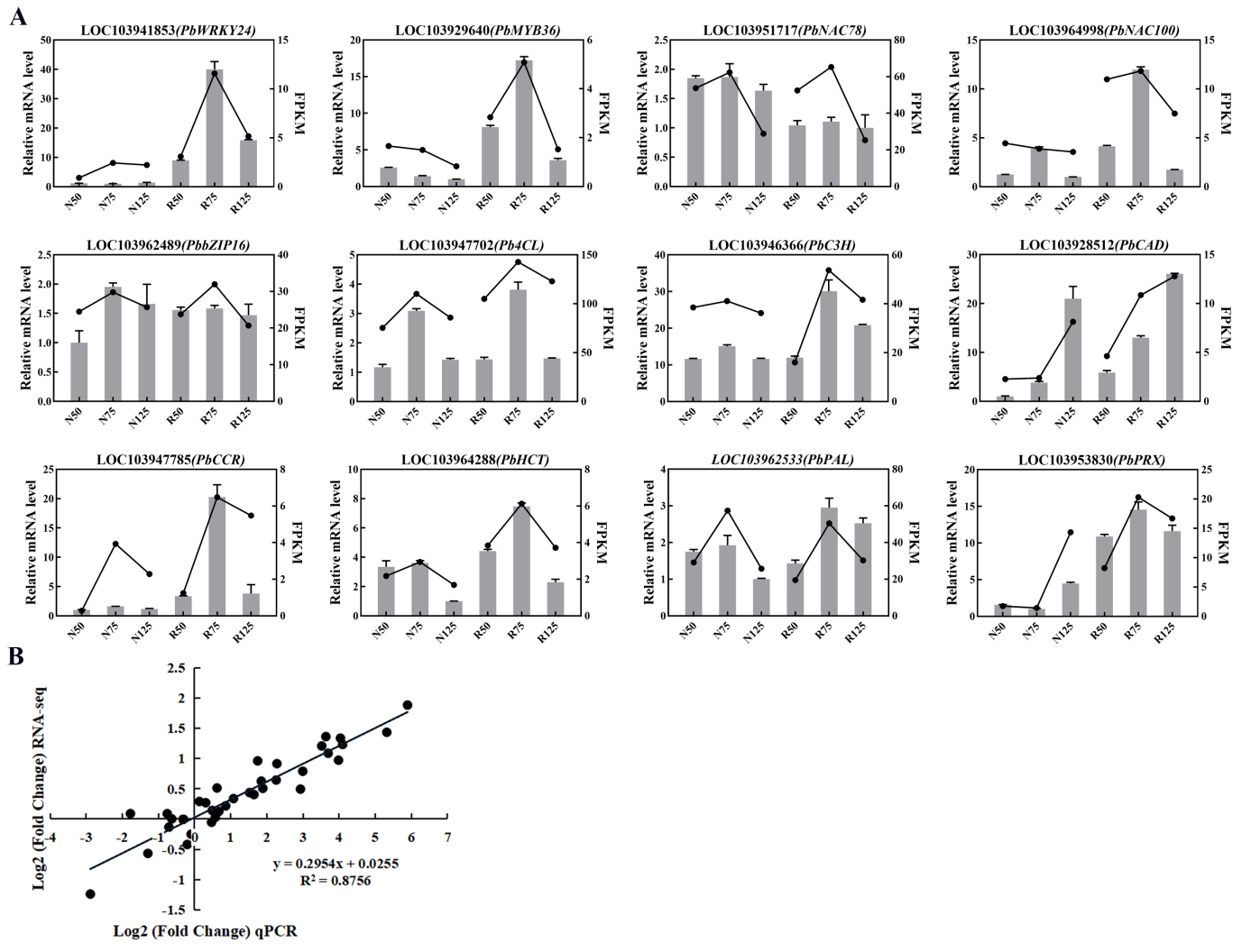


**Figure S2.** Expression levels of differentially expressed genes in pear skin of russet (R) and non-russet skin (N) fruits. (**A**) Quantitative real-time PCR (qPCR) results of 12 selected genes and their RNA sequencing (RNA-seq) data. The left *y*-axis indicates the transcript levels detected by RNA-seq (gray histogram), and the right *y-*axis shows the gene expression levels measured by qPCR (black lines). The *x*-axis represents the time (days) after full bloom. (**B**) Comparison between the log_2_ values of gene expression ratios based on RNA-seq and qPCR.

**
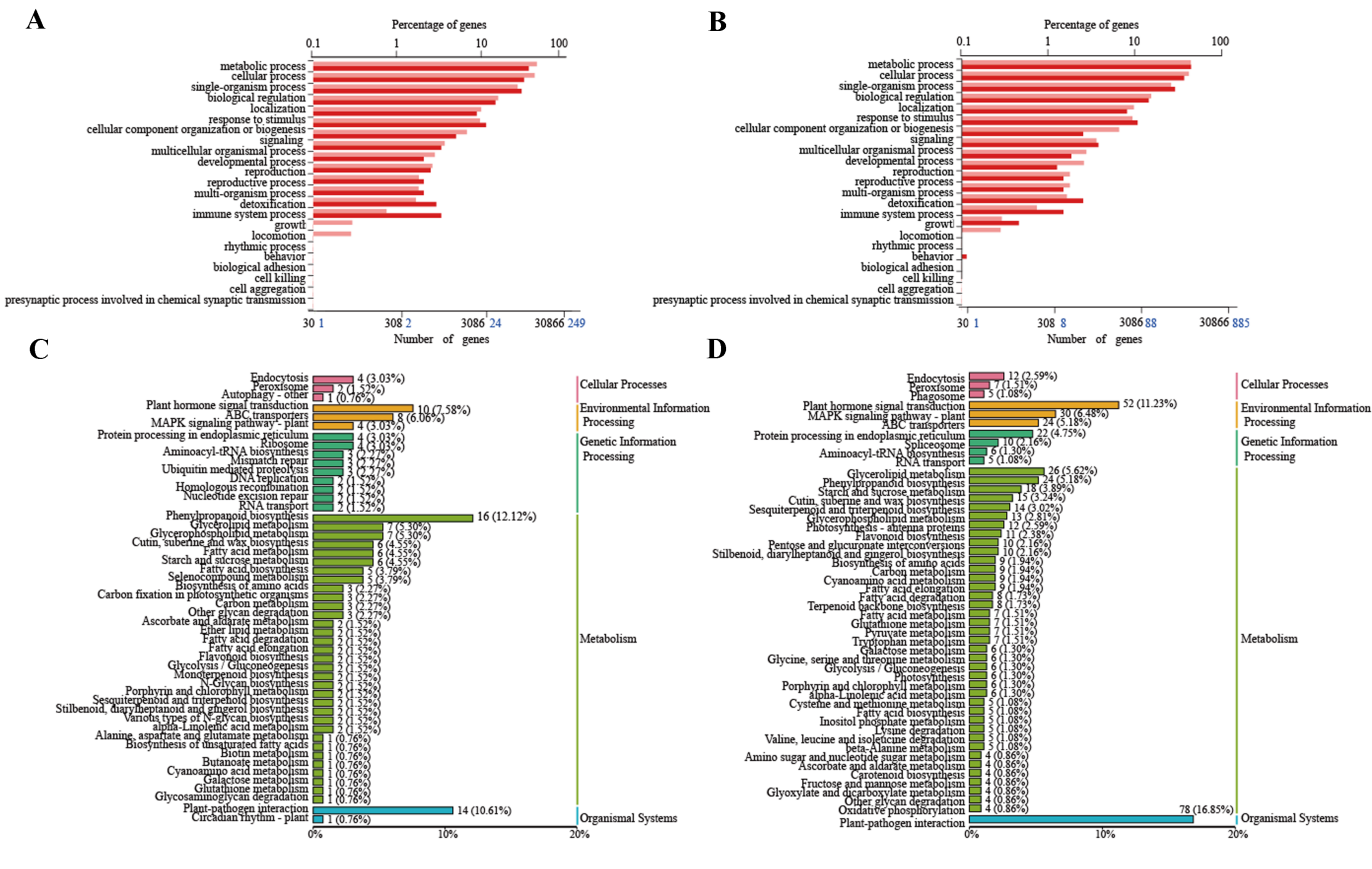
**

**Figure S3.** Functional and pathway enrichment analysis of differentially expressed genes (DEGs) between russet (R) and non-russet skin fruits (N). GO terms enriched for DEGs between different comparison groups: (**A**) R75 vs. N75 and (**B**) R125 vs. N125. KEGG pathways enriched for DEGs between different comparison groups: (**C**) R75 vs. N75 and (**D**) R125 vs. N125. R/N 75 and 125: russet / non-russet skin fruits at 75 and 125 days after full bloom.


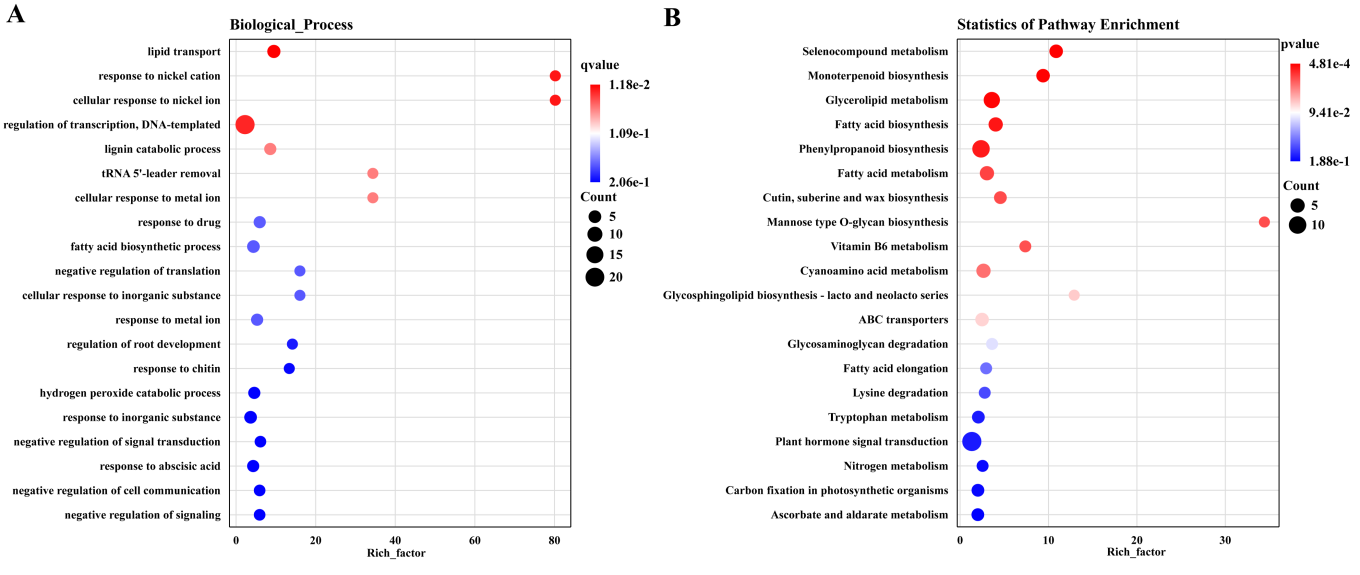


**Figure S4.** The top 20 GO terms (**A**) and KEGG pathways (**B**) enriched for differentially expressed genes in the ‘Paleturquoise’ module.


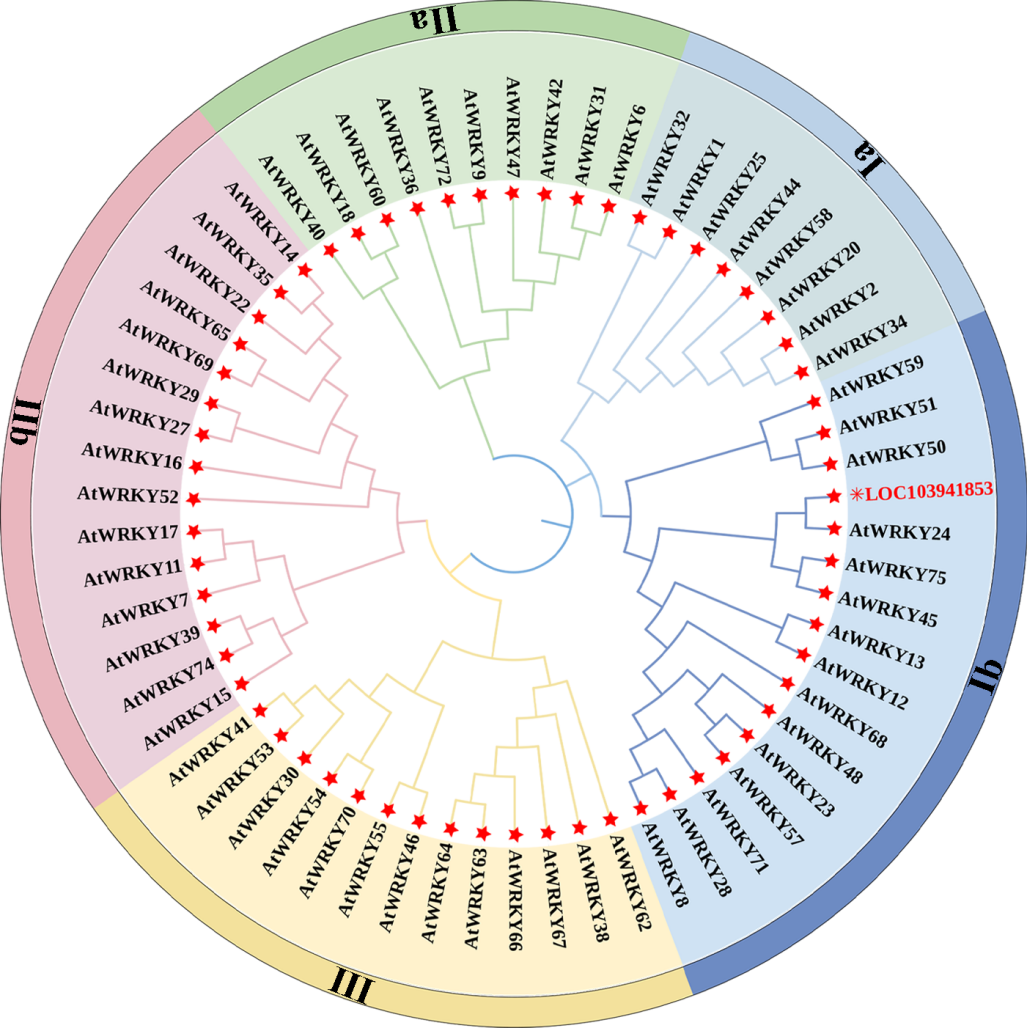


**Figure S5.** Phylogenetic relationships between PpWRKY24 and AtWRKYs. * indicates PpWRKY24.
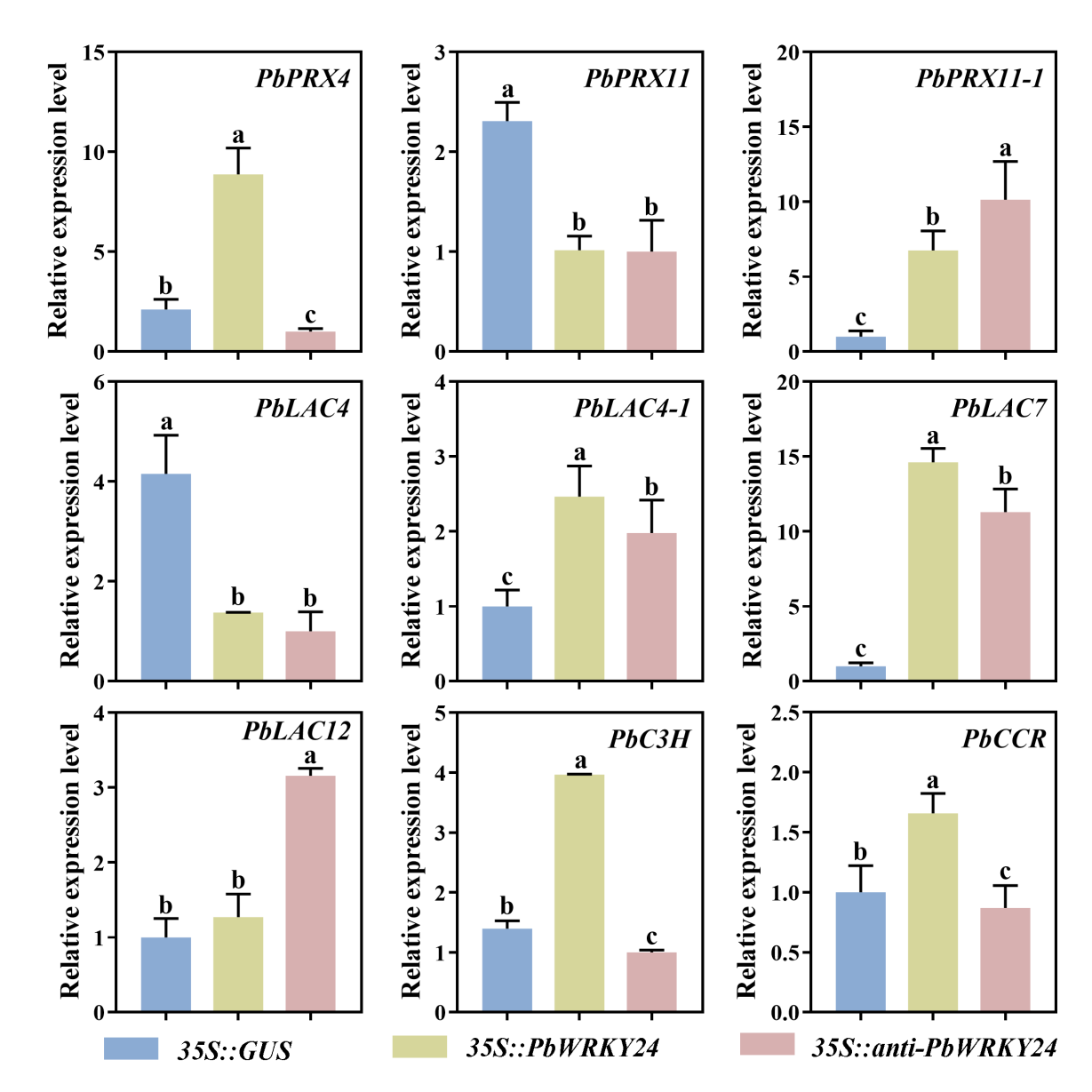


**Figure S6.** Expression patterns of lignin biosynthesis-related genes in transgenic pear fruit skin.


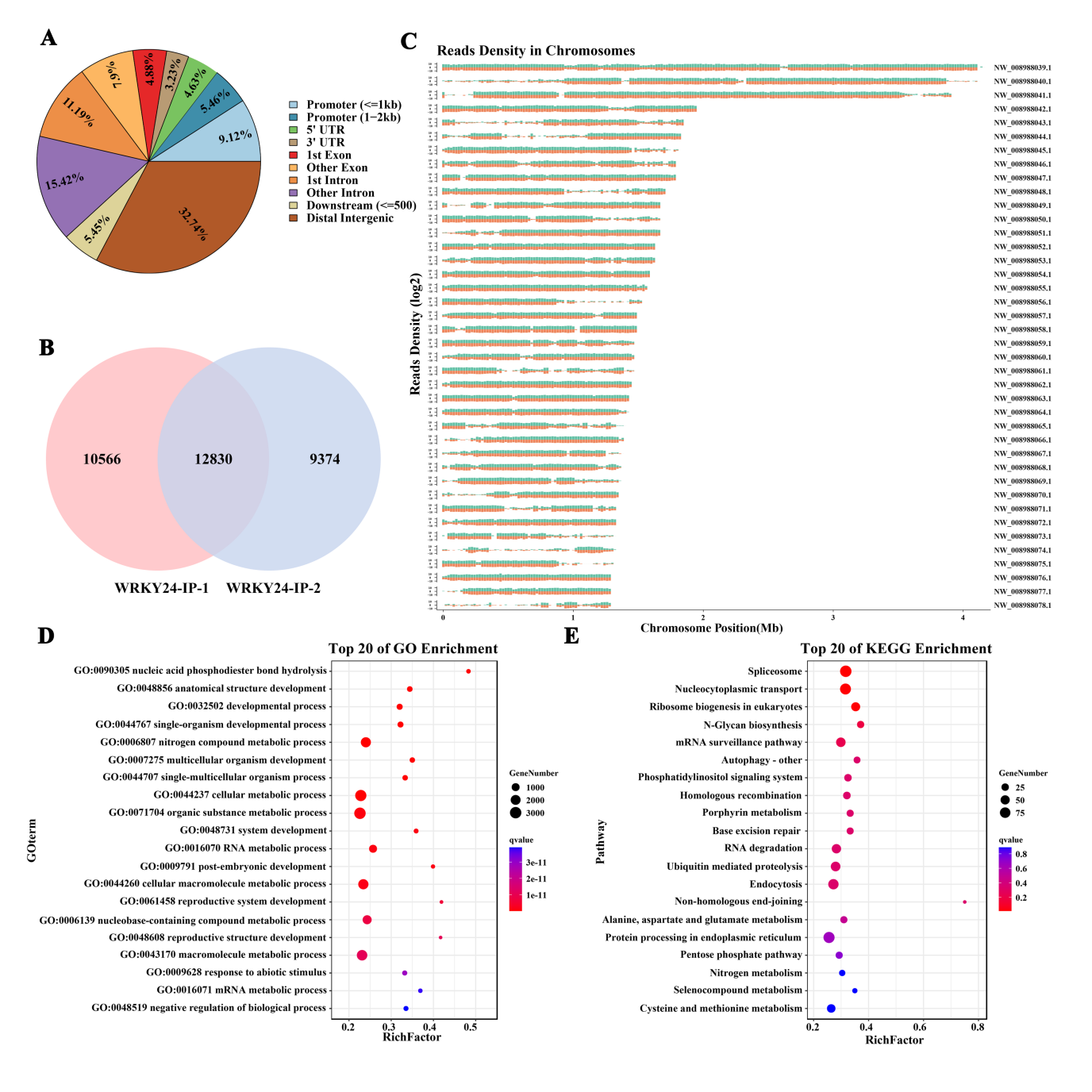


**Figure S7.** Genome-wide analysis of PbWRKY24-binding sites in the pear genome. (A) Identification of PbWRKY24-enriched regions based on DNA affinity purification sequencing. (**B**) Venn diagram showing the unique and shared PbWRKY24-binding peaks between two technical replicate experiments. (**C**) Distribution of PbWRKY24-binding peaks on different chromosomes. (**D**) GO and (**E**) KEGG analysis of the candidate target genes bound by PbWRKY24.


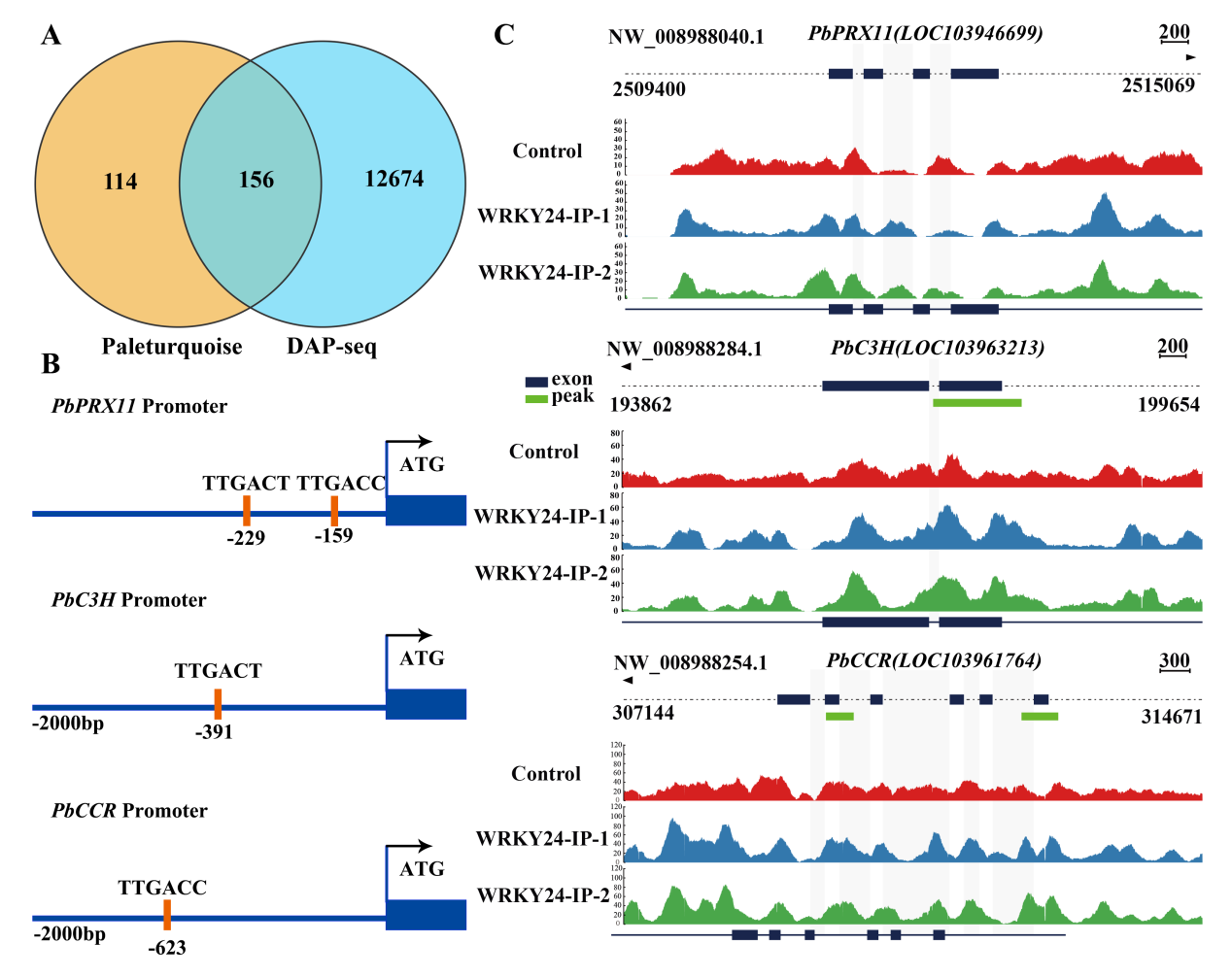


**Figure S8.** Identification of potential target genes of PbWRKY24 based on DNA affinity purification sequencing and RNA sequencing. (**A**) Overlapping of genes between the ‘Paleturquoise’ module and DAP sequencing results. (**B**) Schematic diagrams illustrating the distribution of W-box in the promoter of *PbC3H*, *PbCCR*, and *PbPRX11*. (**C**) Visualization and analysis of *PbC3H*, *PbCCR*, and *PbPRX11* as potential target genes of PbWRKY24.


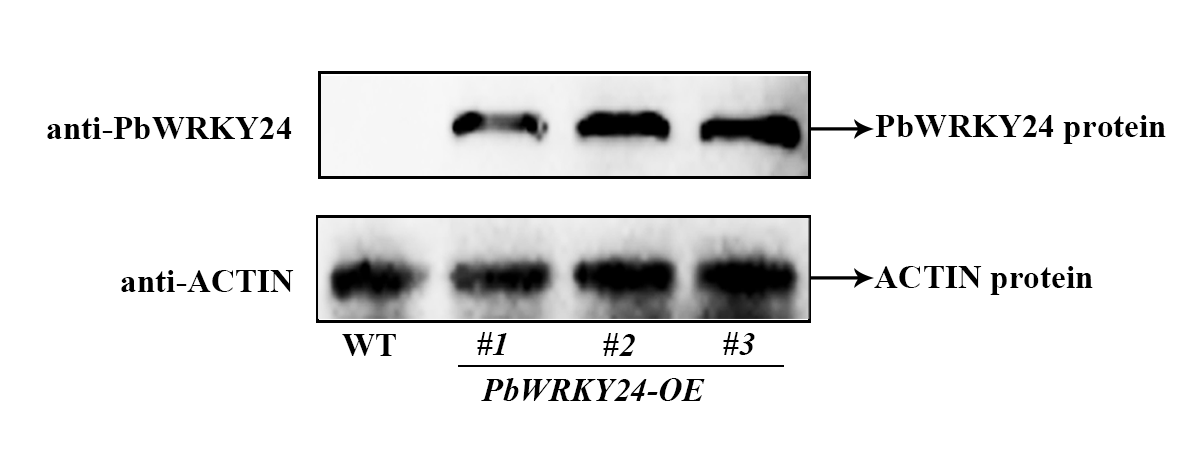


**Figure S9.** Western blotting analysis of PbWRKY24 protein in the overexpressing *PbWRKY24* transgenic tobacco and wild-type tobacco. The PbWRKY24 antibody was used to detect the PbWRKY24 protein of the transgenic tobacco. The ACTIN protein was as control. WT, wild-type tobacco. *PbWRKY24-OE#1*, *#2*, *#3*, overexpressing *PbWRKY24* transgenic tobacco lines1, 2, and 3.
