# Supplementary figures and images for: A transcription factor, PbWRKY24, contributes to russet skin formation in pear fruits by modulating lignin accumulation
Source: Hortic Res. 2024 Oct 18;12(2):uhae300. doi: 10.1093/hr/uhae300 (PMC11822408; doi:10.1093/hr/uhae300)

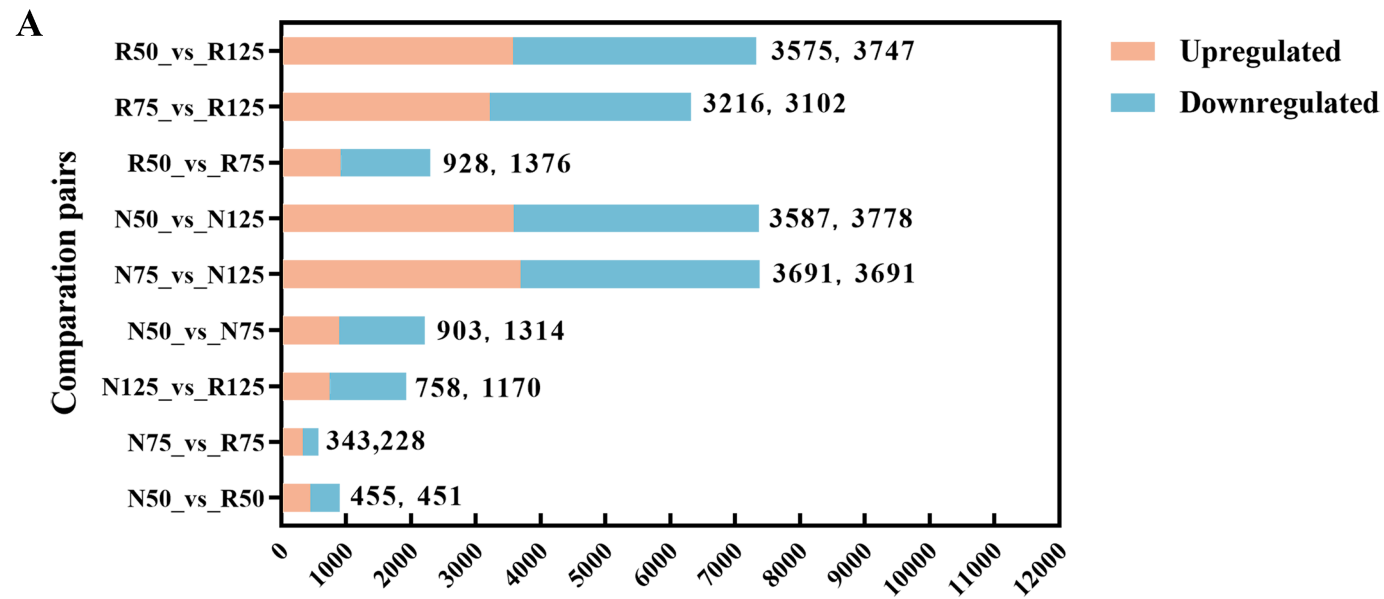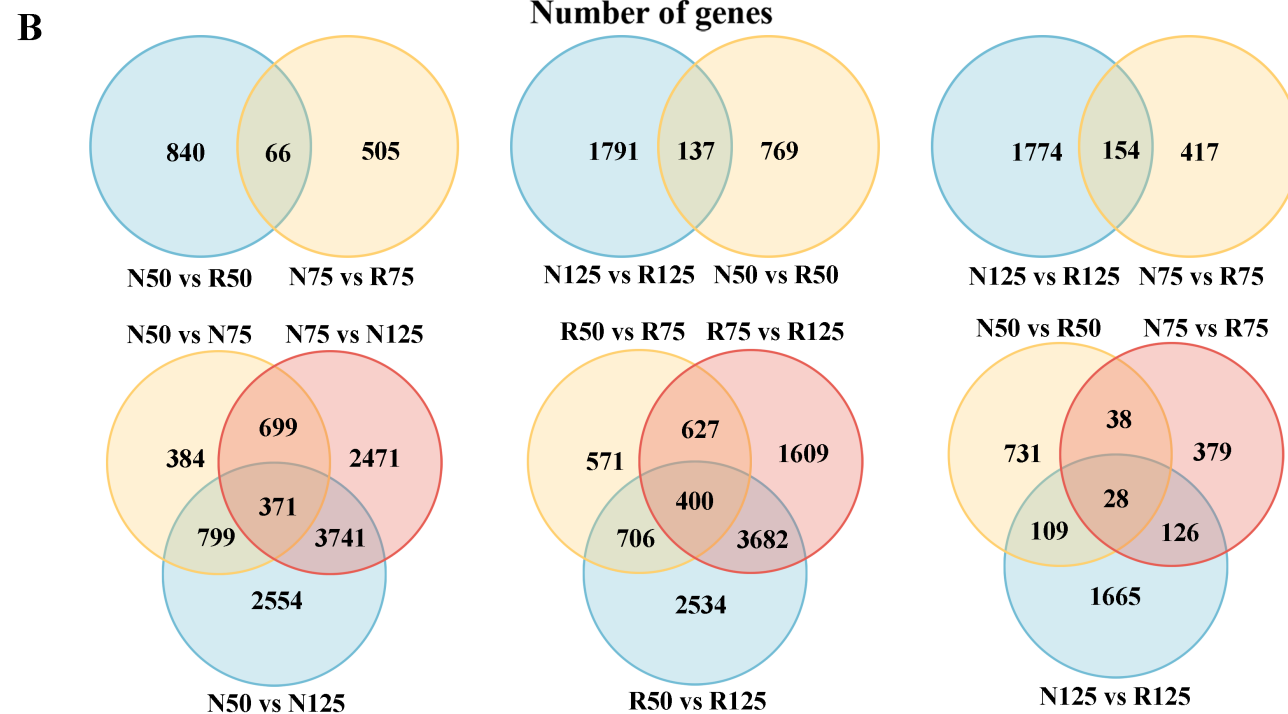

Supplement: Web_Material_uhae300 [file web_material_uhae300.zip › Figure S1.pdf]

**A**

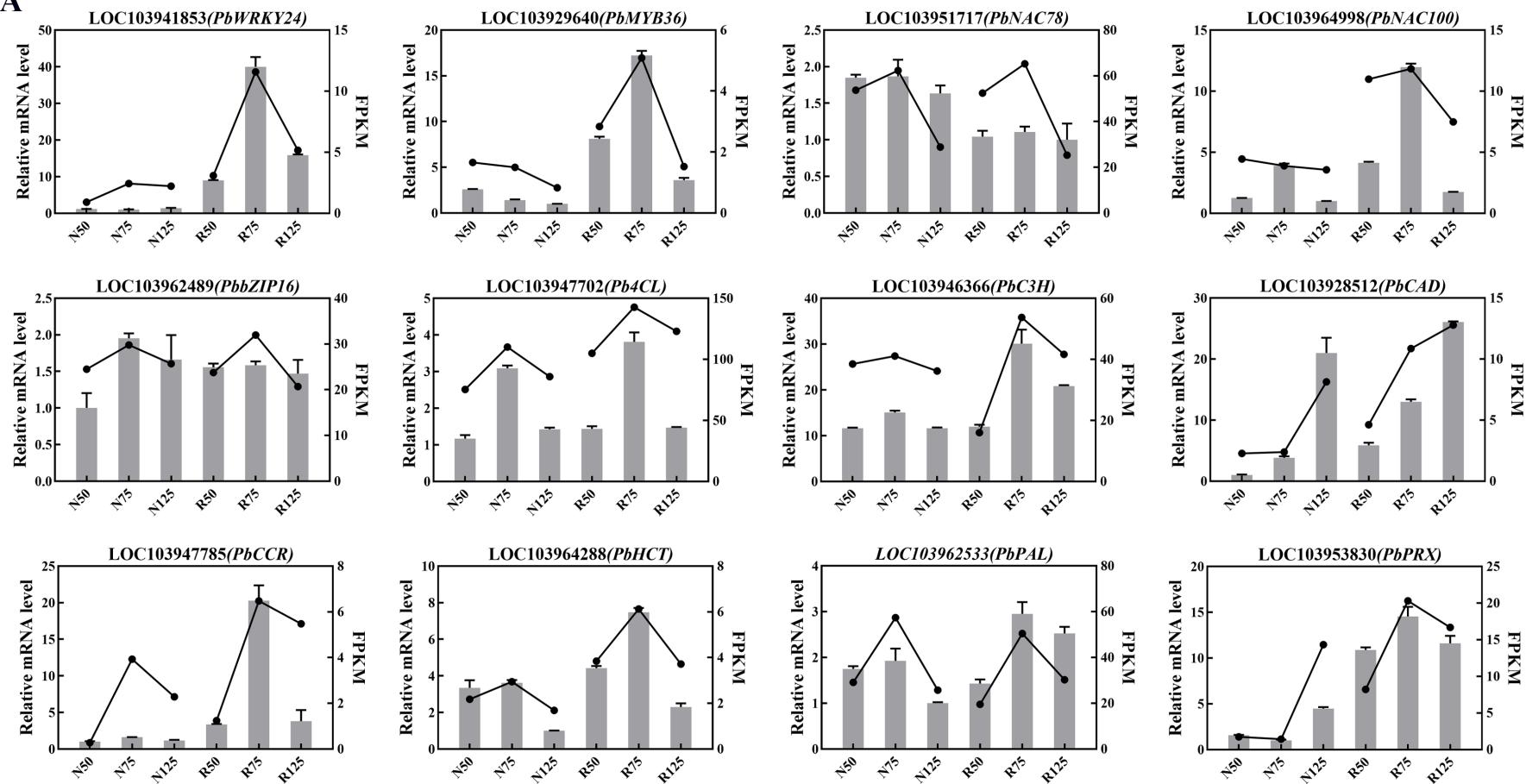

**B**

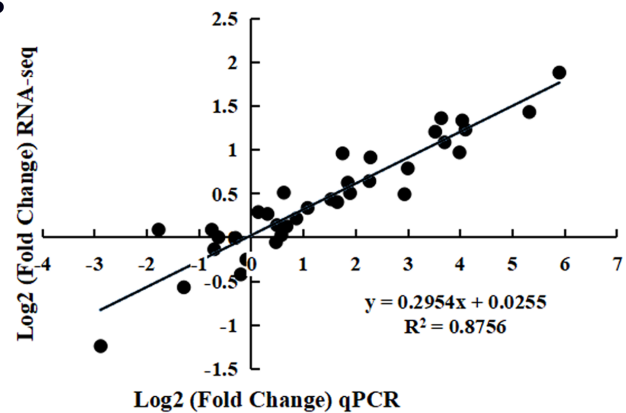

Supplement: Web_Material_uhae300 [file web_material_uhae300.zip › Figure S2.pdf]

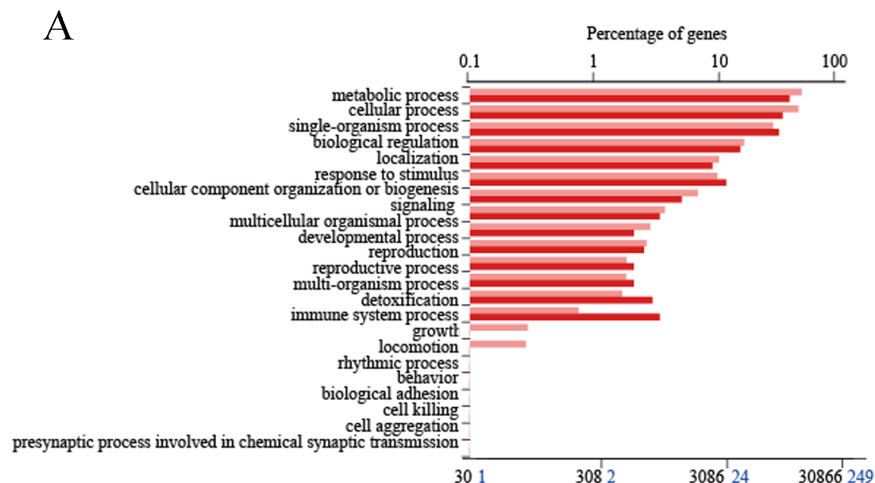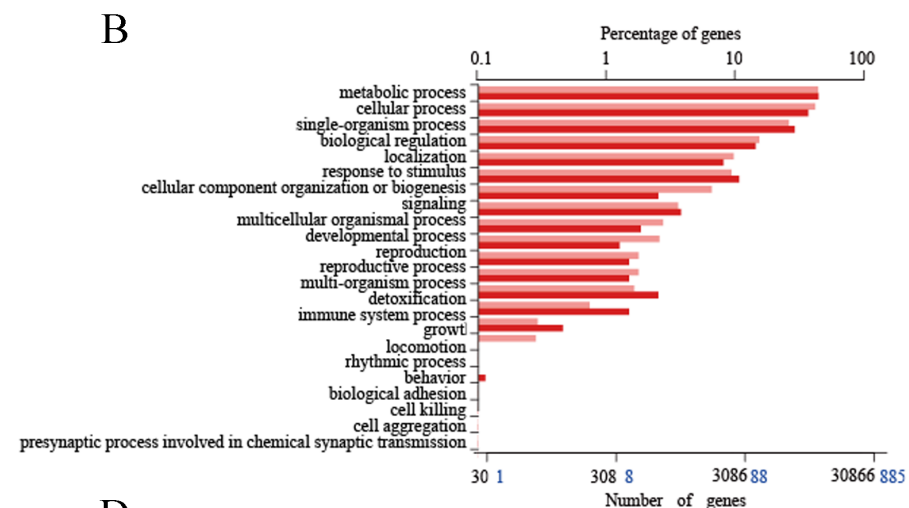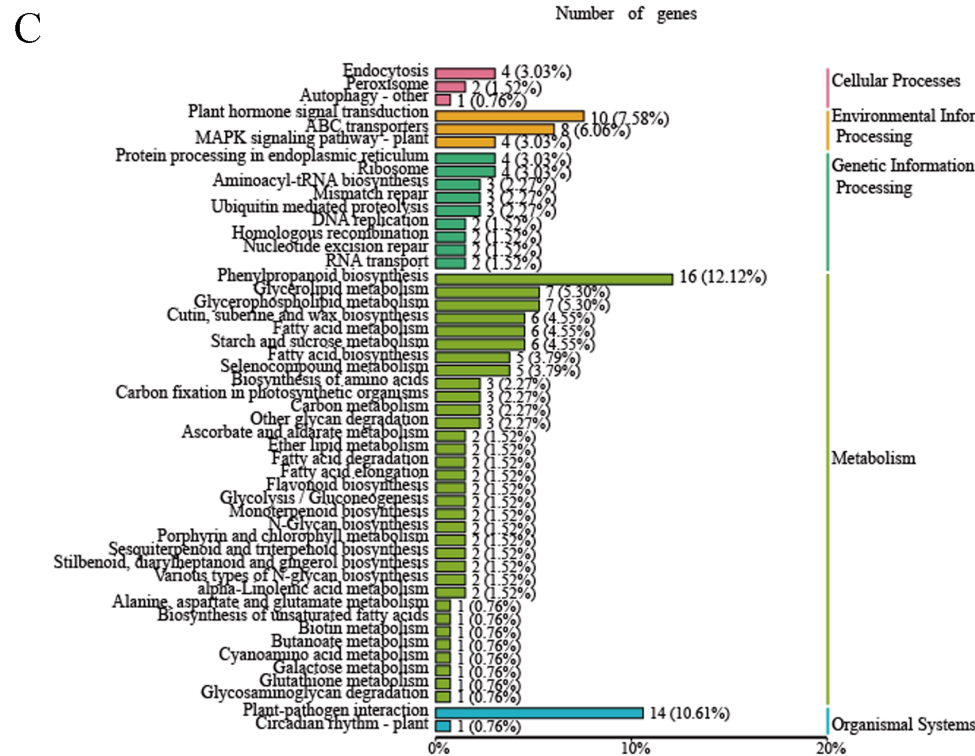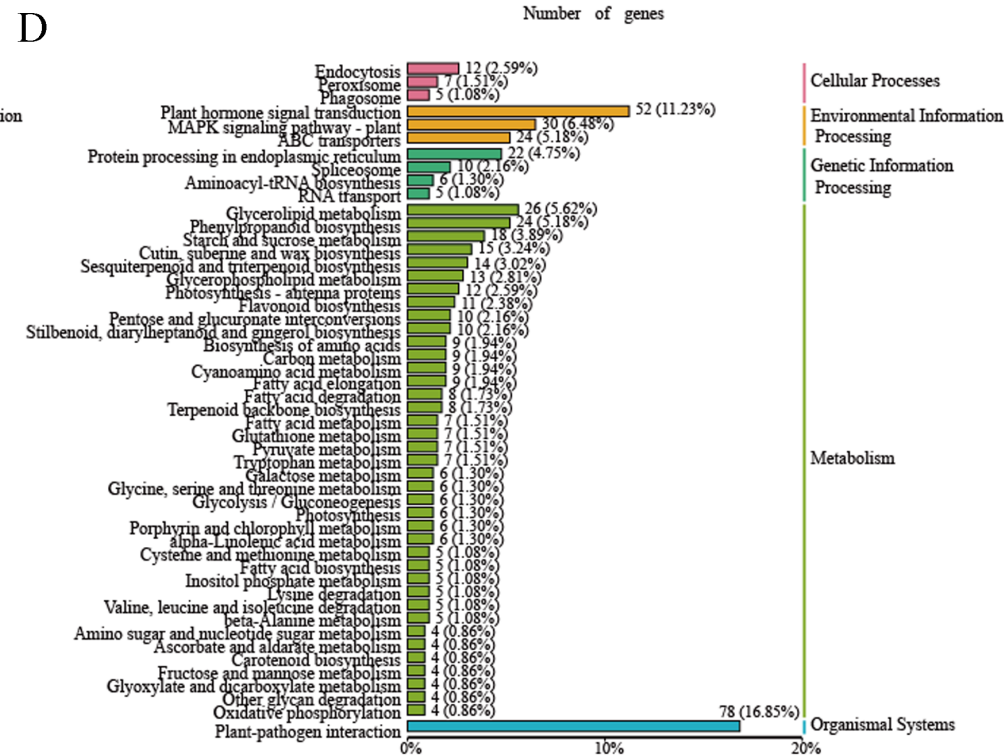

Supplement: Web_Material_uhae300 [file web_material_uhae300.zip › Figure S3.pdf]

A

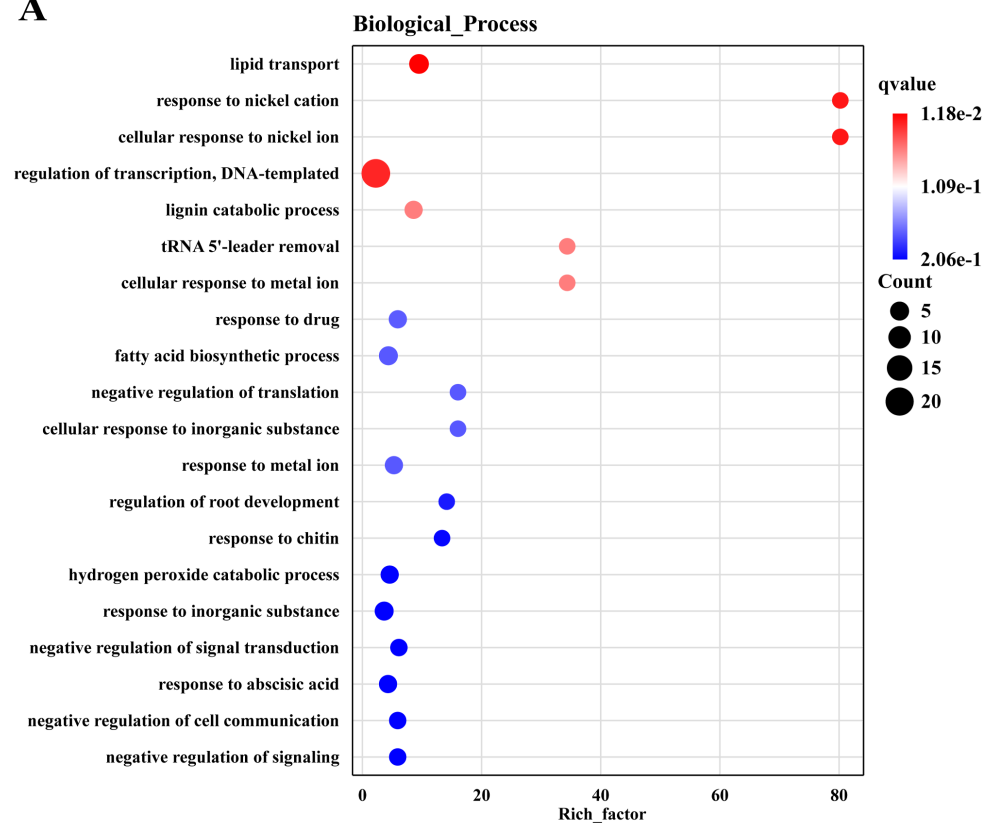

B

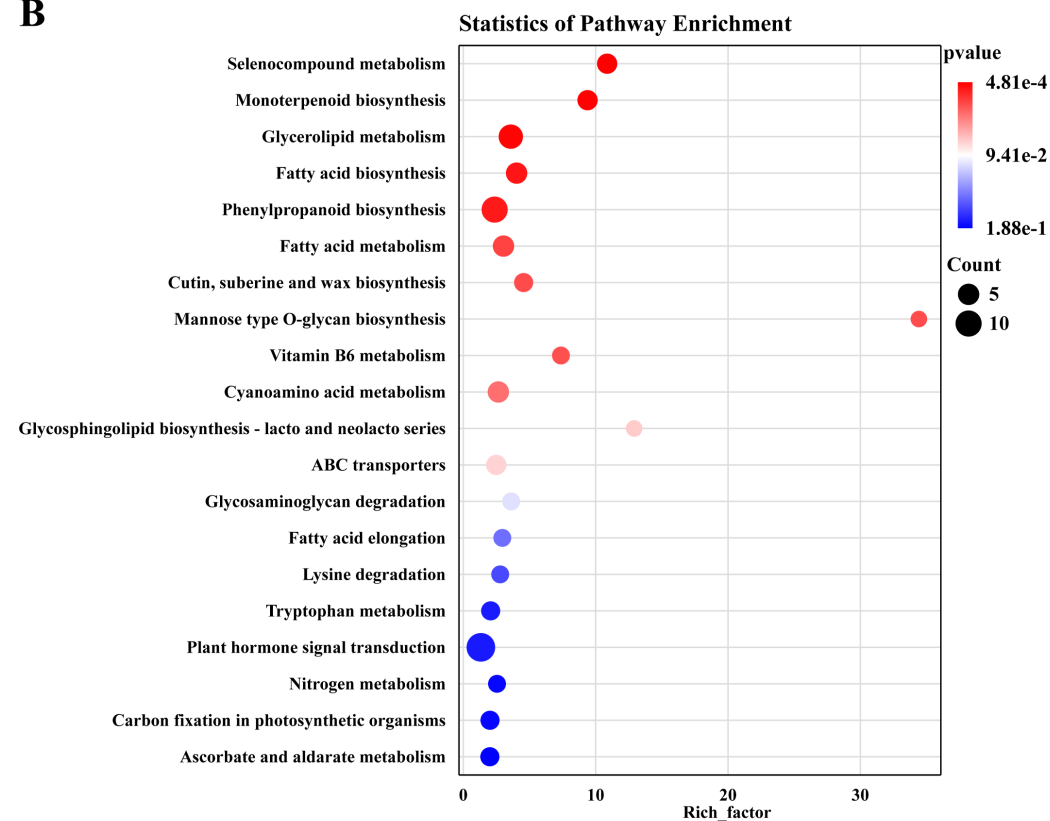

Supplement: Web_Material_uhae300 [file web_material_uhae300.zip › Figure S4.pdf]

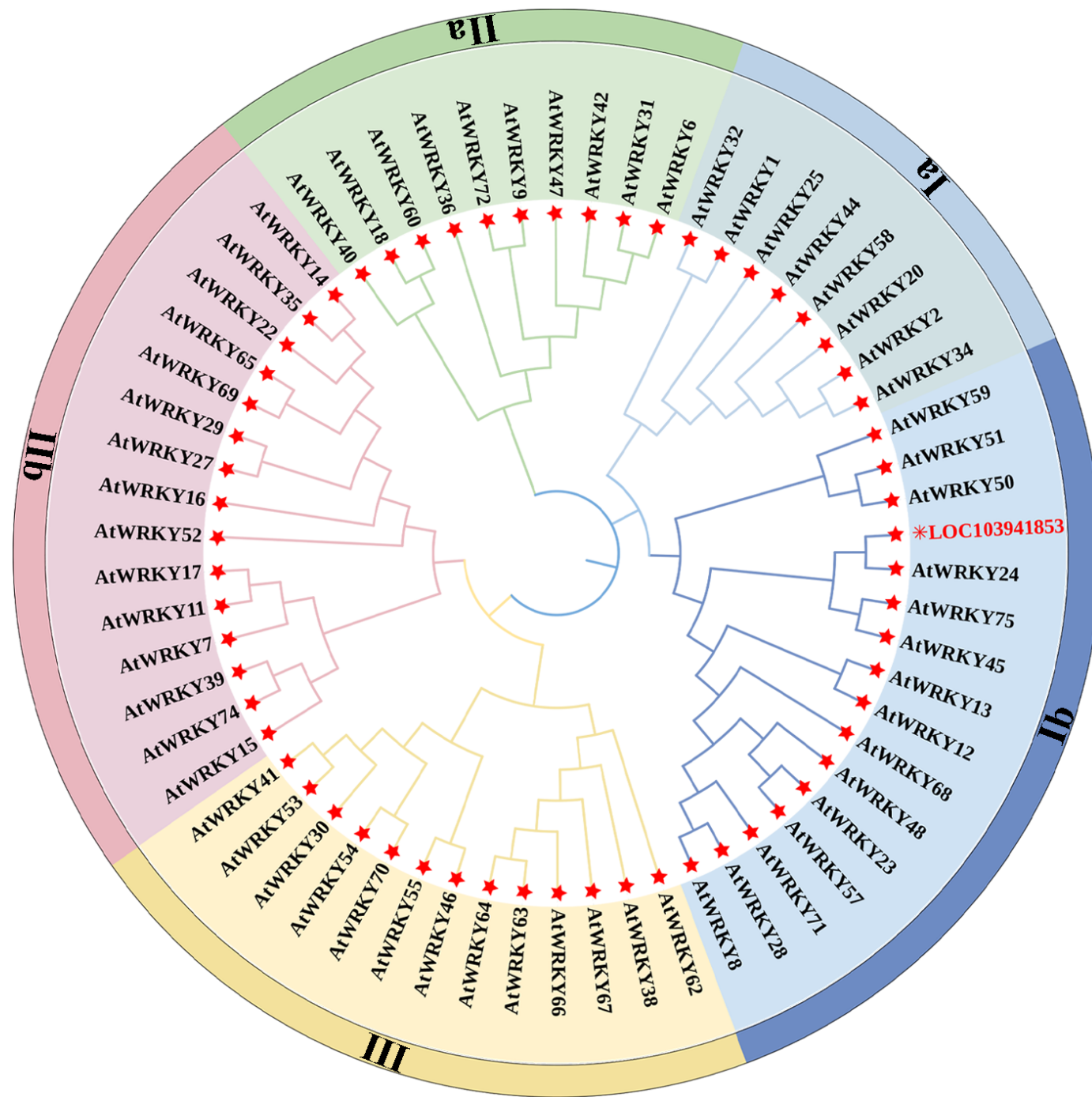

Supplement: Web_Material_uhae300 [file web_material_uhae300.zip › Figure S5.pdf]

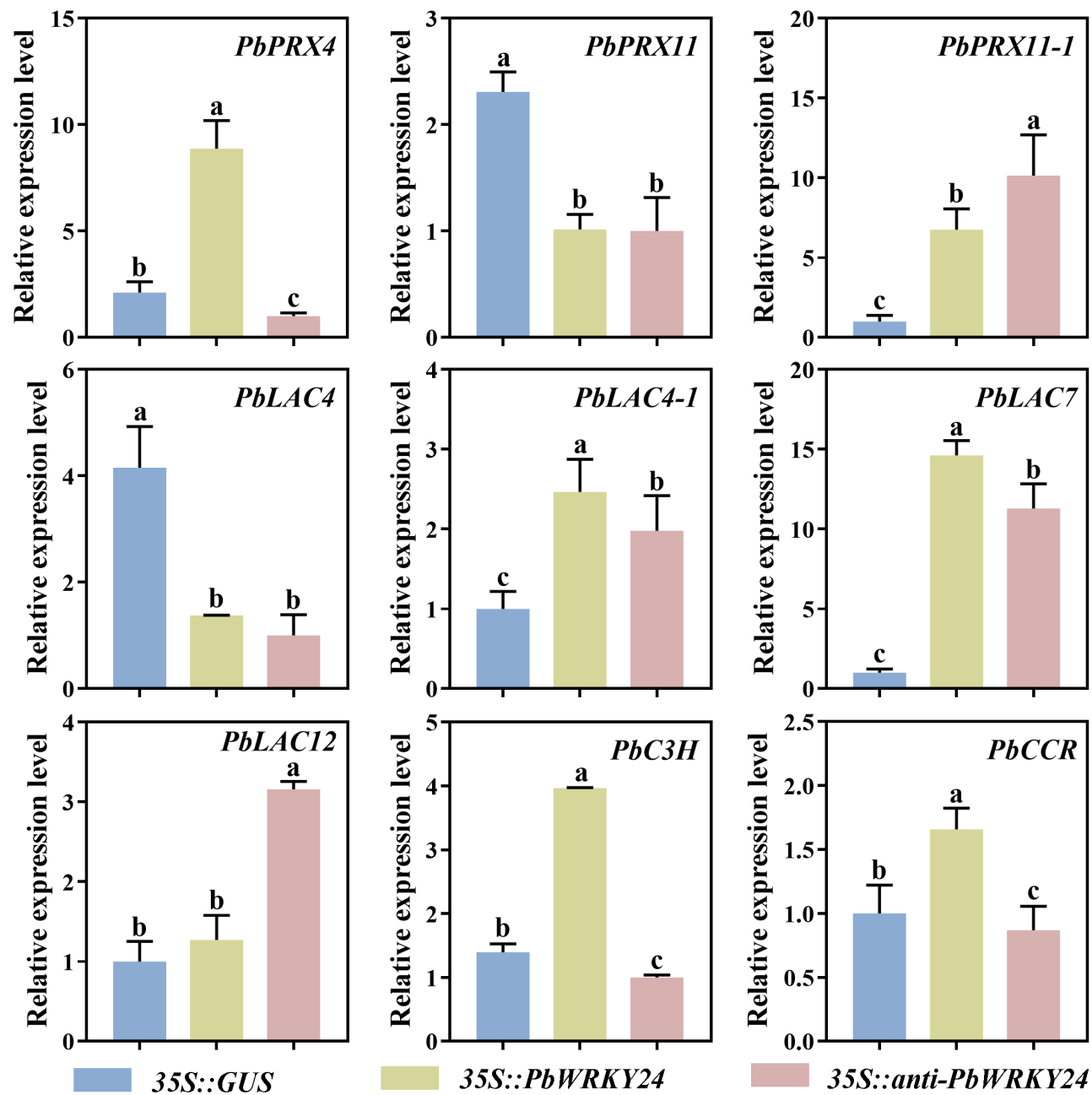

Supplement: Web_Material_uhae300 [file web_material_uhae300.zip › Figure S6.pdf]

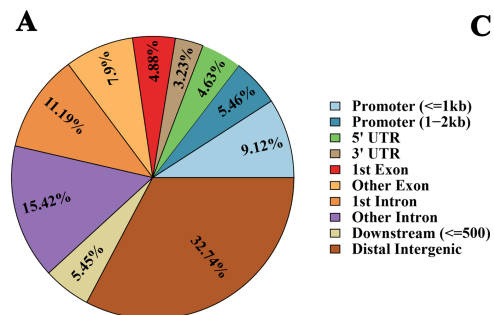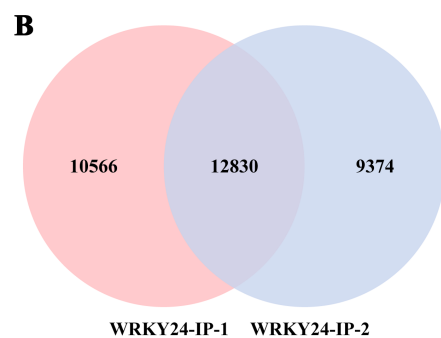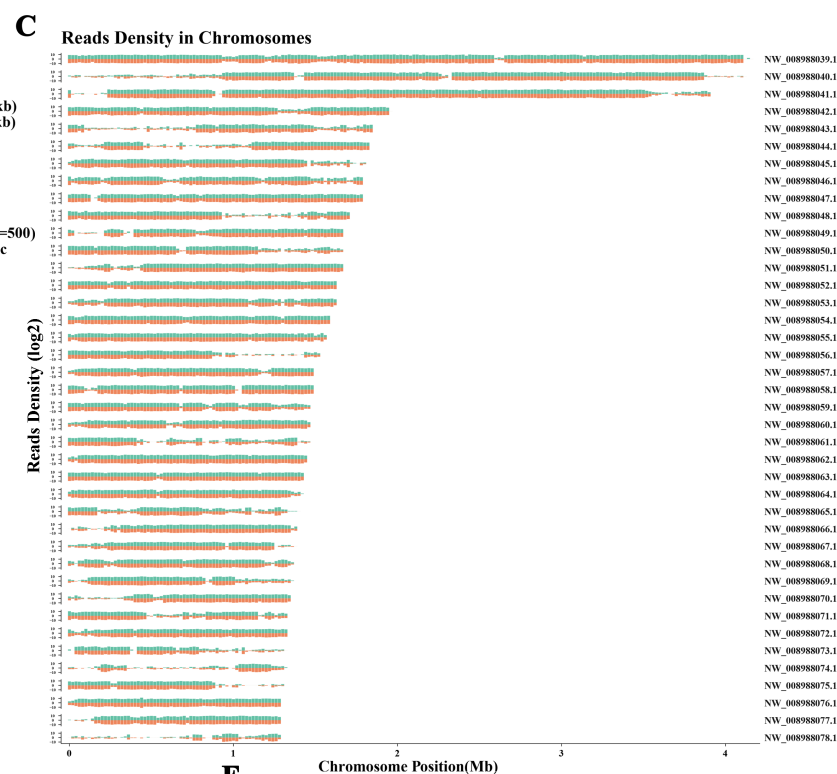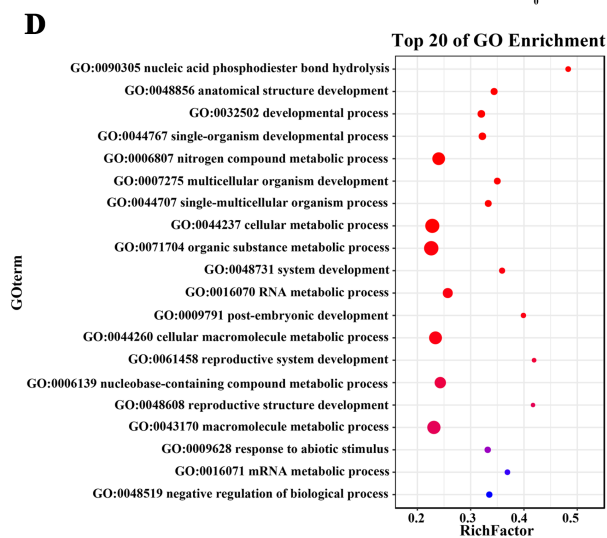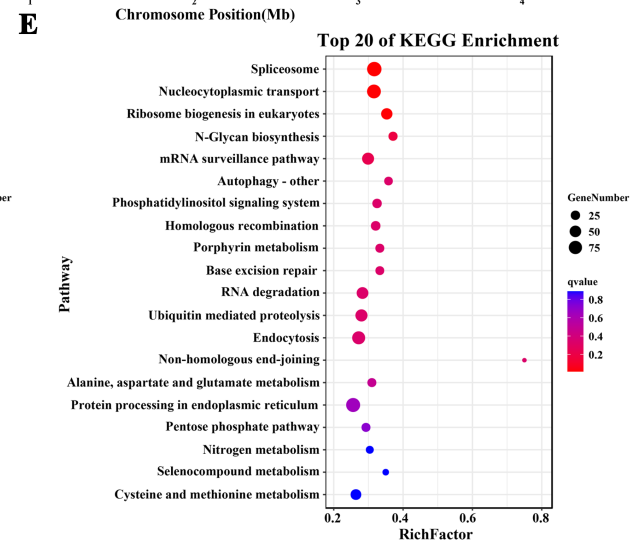

Supplement: Web_Material_uhae300 [file web_material_uhae300.zip › Figure S7.pdf]

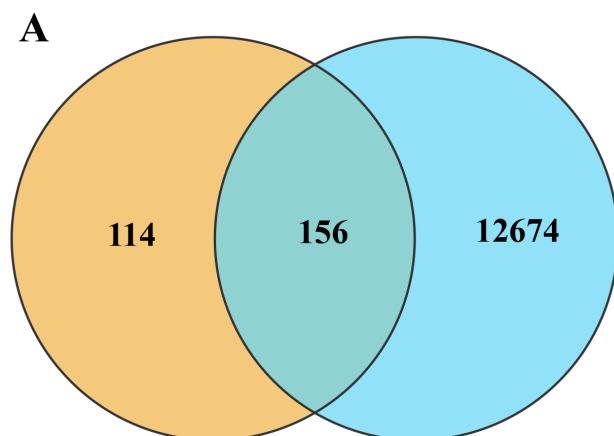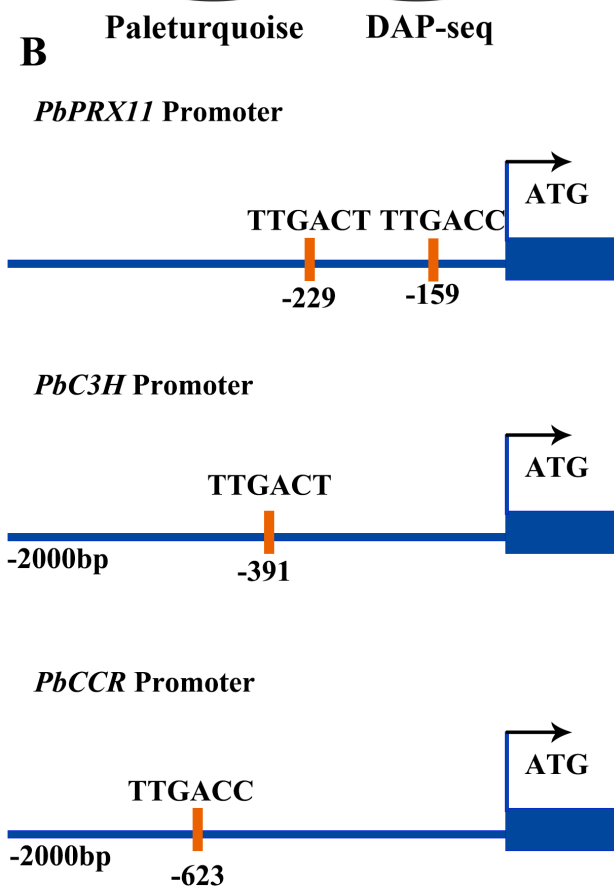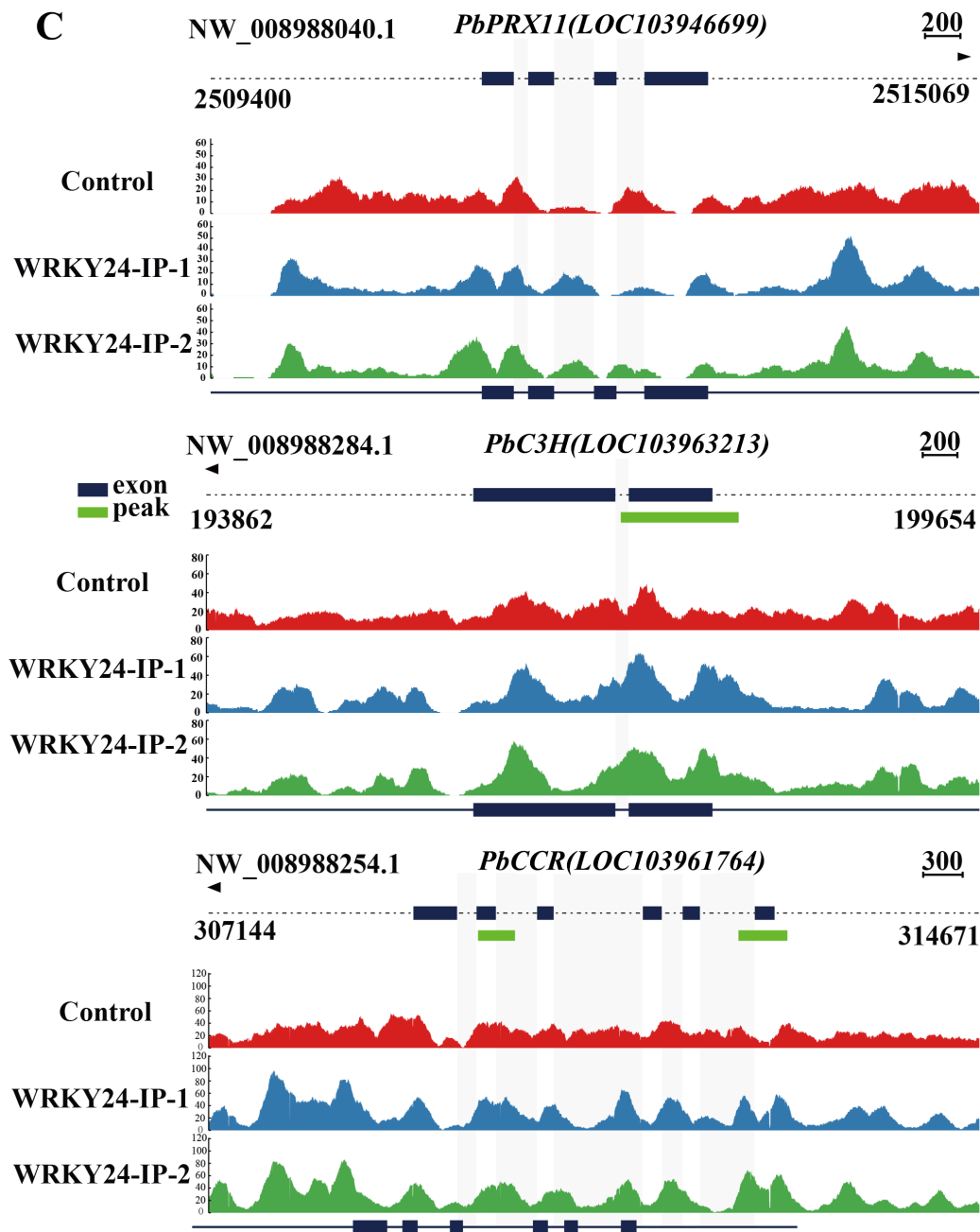

Supplement: Web_Material_uhae300 [file web_material_uhae300.zip › Figure S8.pdf]

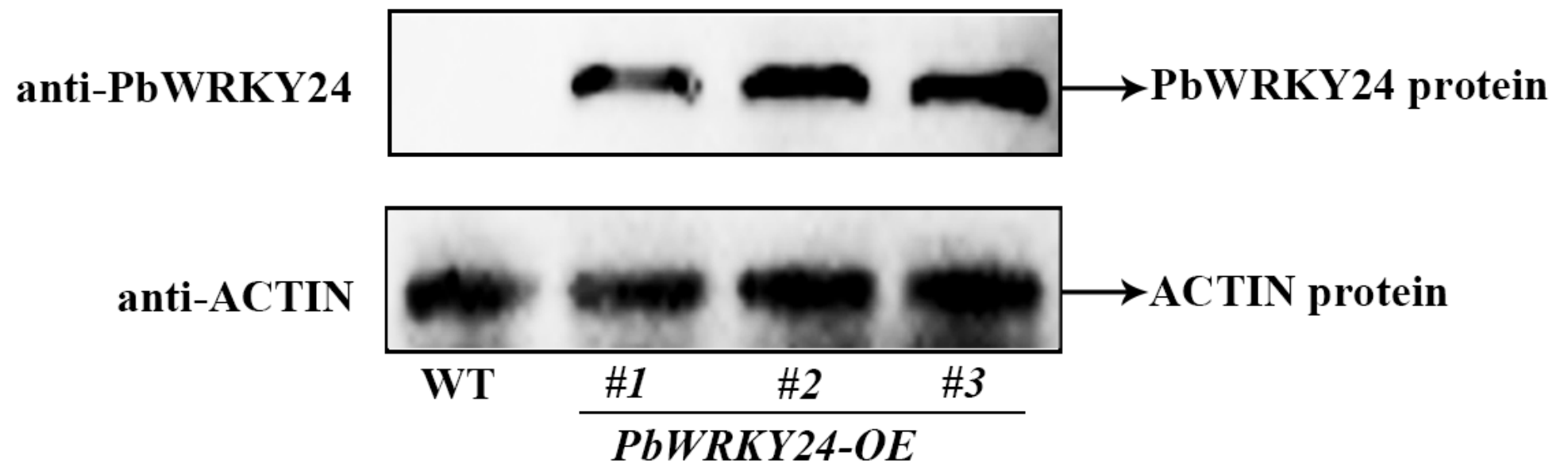

Supplement: Web_Material_uhae300 [file web_material_uhae300.zip › Figure S9.pdf]
